# Fortilin interacts with TGF-β1 and prevents TGF-β receptor activation

**DOI:** 10.1038/s42003-022-03112-6

**Published:** 2022-02-23

**Authors:** Decha Pinkaew, Erik Martinez-Hackert, Wei Jia, Matthew D. King, Fei Miao, Nicole R. Enger, Runglawan Silakit, Kota Ramana, Shi-You Chen, Ken Fujise

**Affiliations:** 1 Division of Cardiology, Department of Medicine, University of Washington, Seattle, WA 98109 USA; 2 Department of Biochemistry and Molecular Biology, Michigan State University, East Lansing, MI 48824 USA; 3 Department of Surgery, University of Missouri, Columbia, MO 65212 USA; 4 Department of Chemistry and Biochemistry, Boise State University, Boise, ID 83725 USA; 5 Department of Biochemistry, Noorda College of Osteopathic Medicine, Provo, UT 84606 USA; 6 Present Address: Department of Pathology and Laboratory Medicine, University of Pennsylvania, Philadelphia, PA 19104 USA

**Keywords:** Proteins, Cell signalling

## Abstract

Fortilin is a 172-amino acid multifunctional protein present in both intra- and extracellular spaces. Although fortilin binds and regulates various cellular proteins, the biological role of extracellular fortilin remains unknown. Here we report that fortilin specifically interacts with TGF-β1 and prevents it from activating the TGF-β1 signaling pathway. In a standard immunoprecipitation-western blot assay, fortilin co-immunoprecipitates TGF-β1 and its isoforms. The modified ELISA assay shows that TGF-β1 remains complexed with fortilin in human serum. Both bio-layer interferometry and surface plasmon resonance (SPR) reveal that fortilin directly bind TGF-β1. The SPR analysis also reveals that fortilin and the TGF-β receptor II (TGFβRII) compete for TGF-β1. Both luciferase and secreted alkaline phosphatase reporter assays show that fortilin prevents TGF-β1 from activating Smad3 binding to Smad-binding element. Fortilin inhibits the phosphorylation of Smad3 in both quantitative western blot assays and ELISA. Finally, fortilin inhibits TGFβ-1-induced differentiation of C3H10T1/2 mesenchymal progenitor cells to smooth muscle cells. A computer-assisted virtual docking reveals that fortilin occupies the pocket of TGF-β1 that is normally occupied by TGFβRII and that TGF-β1 can bind either fortilin or TGFβRII at any given time. These data support the role of extracellular fortilin as a negative regulator of the TGF-β1 signaling pathway.

## Abstract (web summary)

Fortilin prevents the activation of the TGF-β1 receptor by occupying the pocket of TGF-β1 and competing with TGF-βRII to bind with TGF-β1. This inhibits Smad3 phosphorylation and the differentiation of C3H10T1/2 mesenchymal progenitor cells to smooth muscle cells.

## Introduction

Originally cloned in 1988 by Gross et al^1^. as a cytosolic molecule abundantly expressed in tumor cells, fortilin remained a protein of unknown function until 2001 when we^2^ and others^3,4^ reported that it protects against apoptosis. Investigation over the next 20 years revealed that fortilin is a multi-functional protein implicated in diverse biological processes, including protection against apoptosis^2–4^, endoplasmic reticulum stress handling^5^, cell cycle progression^6^, reactive oxygen species detoxification^7^, and Ig-E-mediated histamine release^8,9^. Fortilin exists in both the cytosol and the nucleus (cellular fortilin)^2^ and circulates in the blood^10^ after being secreted from the cell (circulating fortilin)^11^.

In the cell, fortilin exerts its biological activities through its molecular interaction with its “executioner” proteins. For example, fortilin binds and negatively regulates p53 by preventing p53 from transcriptionally activating its target molecules BAX, PUMA, and NOXA^12,13^. In addition, fortilin binds and positively regulates peroxiredoxin-1, a reactive oxygen species-detoxifying enzyme, by preventing it from being inactivated by Mst1 kinase^7^. Moreover, fortilin binds and stabilizes the myeloid cell leukemia protein-1 (MCL1), a Bcl-2 family member anti-apoptotic molecule, thereby supporting the survival of myeloid cells^14,15^. Finally, fortilin binds and inhibits the inositol-requiring enzyme 1 alpha (IRE1α), an endoplasmic reticulum (ER) stress sensor^5^.

We and others have shown that fortilin is secreted from the cell^10^ via the non-classical pathway^11^ and circulates in the blood of humans and mice^10^. However, the biological activity of circulating fortilin has been poorly understood. Herein we report that fortilin physically binds TGF-β1, functionally inhibits the TGF-β1 signaling pathway, and prevents mesenchymal progenitor cells from differentiating into vascular smooth muscle cells. We propose that extracellular fortilin is an inhibitor of TGF-β1.

## Results

### Co-immunoprecipitation (Co-IP) assays showed that fortilin binds TGF-β1

**Fig. 1:**
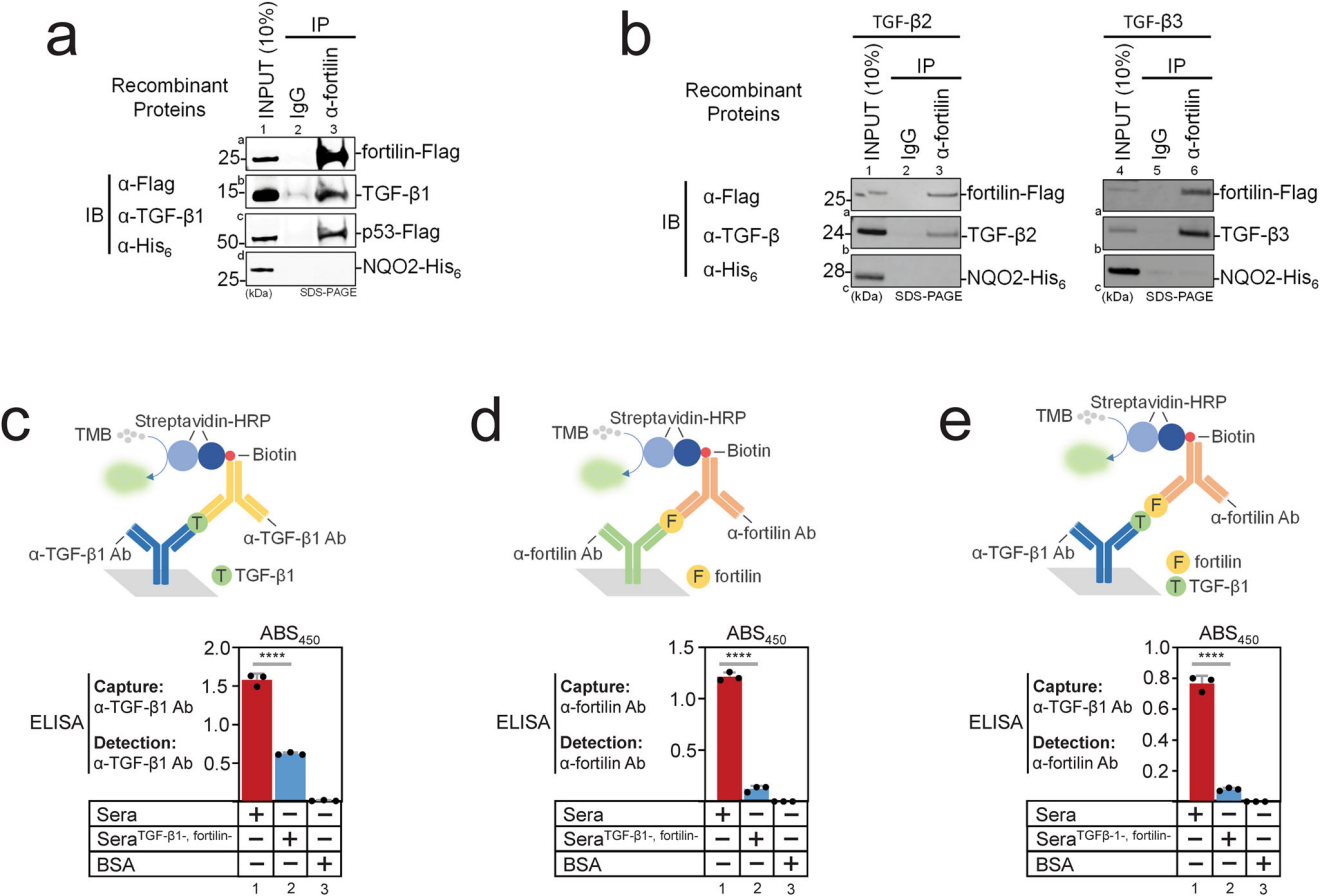
Fortilin and TGF-βs specifically interact with each other. IP, immunoprecipitation; IB, immunoblot; α-Flag, anti-FLAG antibody; α-TGF-β1, anti-TGF-β1 antibody; α-His_6_, anti-hexa-histidine antibody; ELISA, enzyme-linked immunosorbent assay; TMB, 3,3’,5,5’-tetramethylbenzidine; HRP, horse-radish peroxidase; Capture, capturing antibody; Detection, detecting antibody; Sera^TGF-β1+, fortilin+^, sera generated from platelet-rich plasma; Sera^TGF-β1-, fortilin-^, Sera^TGF-β1+, fortilin+^ immunodepleted of both fortilin and TGF-β1; BSA, bovine serum albumin; Ab, antibody; ABS_450_, absorbance at 450 nm; Data points, means ± SD; statistical analyses performed using ANOVA with Fisher’s multiple comparison; ****P < 0.001. **a** Fortilin interacts with TGF-β1. Recombinant Flag-tagged fortilin, TGF-β1, Flag-tagged p53, and His_6_-tagged NQO2 were incubated in binding buffer. α-fortilin antibody was used to immunoprecipitate fortilin, and immune complexes were resolved in SDS-PAGE and blotted onto a nitrocellulose membrane. Immunoblot analyses using α-TGF-β1, α-Flag, and α-His_6_ antibodies showed that fortilin co-immunoprecipitated TGF-β1 and p53 but not NQO2. Fortilin is known to interact with p53 but not with NQO2. INPUT represented 10% of the total reaction mixture used for IP. **b** Fortilin interacts with TGF-β2 and -β3. Recombinant Flag-tagged fortilin, TGF-β2 or TGF-β3, and His_6_-tagged NQO2 were incubated in binding buffer. α-fortilin antibody was used to immunoprecipitate fortilin, and immune complexes were resolved in SDS-PAGE and blotted onto a nitrocellulose membrane. Immunoblot analyses using α-Flag, α-TGF-β, and α-His_6_ antibodies showed that fortilin co-immunoprecipitated TGF-β2 and -β3, but not NQO2. INPUT represented 10% of the total reaction mixture used for IP. **c** Detection of TGF-β1 in normal human serum. The TGF-β1 ELISA that used α-TGF-β1 capturing and detecting Abs showed high TGF-β1 levels in Sera^TGF-β1+, fortilin+^ (column 1) compared with those in Sera^TGF-β1-, fortilin-^ (column 2). *N* = 3. **d** Detection of fortilin in normal human serum. The in-house fortilin ELISA that used α-fortilin capturing and detecting Abs showed high fortilin levels in Sera^TGF-β1+, fortilin+^ (column 1) compared with those in Sera^TGF-β1-, fortilin-^ (column 2). *N* = 3. **e** Detection of the fortilin-TGF-β1 interaction in normal human serum. The modified ELISA system that used α-TGF-β1 capturing and α-fortilin detecting Abs yielded a robust signal in Sera^TGF-β1+, fortilin+^ (column 1) compared with those in Sera^TGF-β1-, fortilin-^ (column 2). *N* = 3.

To test whether fortilin physically interacts with TGF-β1, we performed in vitro Co-IP assays using recombinant human fortilin (rh-fortilin) and TGF-β1 (rh-TGF-β1) proteins. We equally divided the reaction mixture containing rh-fortilin, rh-TGF-β1, rh-p53, and rh-N-Ribosyldihydronicotinamide:Quinone Reductase 2 (rh-NQO2) into two microfuge tubes (Fig. 1a, INPUT; Supplementary Fig. 4 or Fig. S4 hereafter). We added rabbit IgG to the first tube and rabbit monoclonal anti-fortilin antibody (α-fortilin mAb) to the second tube. After incubation and extensive washing, the immune complex pulled down with anti-rabbit-IgG magnetic beads was eluted into SDS-loading buffer. The system was functioning appropriately, as fortilin was able to co-immunoprecipitate p53, a known fortilin-interacting protein^12^ (Fig. 1a, lane 3, row c). Washing was sufficiently stringent to prevent fortilin from nonspecifically binding NQO2, a protein that is known not to interact with fortilin^7^ (Fig. 1a, lane 3, row d). Using this protocol, the presence of co-immunoprecipitated TGF-β1 was evaluated by the same western blot analysis. We found that α-fortilin mAb (Fig. 1a, lane 3, row a), but not IgG (Fig. 1a, lane 2, row a), immunoprecipitated fortilin and that TGF-β1 was successfully co-immunoprecipitated in the presence of fortilin (Fig. 1a, lane 3, row b) but not in its absence (Fig. 1a, lane 2, row b), suggesting that fortilin and TGF-β1 specifically interact with each other.

To test whether fortilin also interacts with other isoforms of TGF-β (TGF-β2 and TGF-β3), we performed the same Co-IP experiment using rh-TGF-β2 and rh-TGF-β3. We found that α-fortilin mAb (Fig. 1b, lane 3, row a; lane 6, row a; Figs. S5, S6), but not IgG (Fig. 1b, lane 2, row a; lane 5, row a), immunoprecipitated fortilin and that fortilin was capable of co-immunoprecipitating both TGF-β2 (Fig. 1b, lane 3, row b) and TGF-β3 (Fig. 1b, lane 6, row b) but not NQO2 (Fig. 1b, lane 3, row c; lane 6, row c). These data suggest that fortilin specifically interacts with TGF-β1, TGF-β2, and TGF-β3.

Fortilin circulates in the mouse and human blood at the serum concentrations of 2.17 ± 0.58 nM (biological replicates (*N*) = 30) and 3.41 ± 2.07 nM (*N* = 63), respectively, as we previously reported^10^. To assess the relative contribution of TGF-β1, TGF-β2, and TGF-β3 to the formation of a complex with fortilin in the blood, we measured their serum concentrations using a multiplex assay system (MILLIPLEX™ MAP TGFβ Magnetic Bead 3 Plex Kit, Millipore Sigma, Burlington, MA). We found the concentrations of TGF-β1, TGF-β2, and TGF-β3 to be 10.5 ± 2.1, 1.2 ± 0.06, and <0.023 (lower than the detection limit) nM, respectively (*N* = 15 each) (Fig. S1a), suggesting that the fortilin-TGF-β1 interaction represents the majority of fortilin-TGF-β interactions in the blood. Similar results have been reported for normal human plasma as well^16,17^. We therefore focused on the fortilin-TGF-β1 interaction in the remainder of the project.

To determine if fortilin and TGF-β1 form a complex in vivo in normal human circulation, we obtained de-identified platelet-rich plasma from the University of Washington Blood Bank and generated serum by adding thrombin to the plasma. We divided the serum into two portions and, from one portion, immunodepleted both fortilin and TGF-β1 using magnetic beads coated with either α-fortilin-mAb or α-TGF-β-mAb (Sera^TGF-β1-, fortilin-^). We then subjected both the original sera (Sera^TGF-β1+, fortilin+^) and Sera^TGF-β1-, fortilin-^ to three distinct ELISA systems (Fig. 1c–e). We first performed standard TGF-β1 ELISA using α-TGF-β1 capturing Ab and α-TGF-β1 detecting Ab. We found that Sera^TGF-β1+, fortilin+^ contained ~2.5 time more TGF-β1 than Sera^TGF-β1-,  fortilin-^ (Sera^TGF-β1+, fortilin+^ vs. Sera^TGF-β1-, fortilin-^ = 1.58 ± 0.078 vs. 0.62 ± 0.017 [Absorbance 450 or Abs_450_]) (Fig. 1c, columns 1 vs. 2). We then performed standard fortilin ELISA as described previously^10^ using α-fortilin capturing Ab and α-fortilin detecting Ab. We found that Sera^TGF-β1+, fortilin+^ contained ~10 times more fortilin than Sera^TGF-β1-, fortilin-^ (Sera^TGF-β1+, fortilin+^ vs. Sera^TGF-β1-, fortilin-^ = 1.21 ± 0.042 vs. 0.12 ± 0.029 Abs_450_) (Fig. 1d, columns 1 vs. 2). When taken together (Fig. 1c, d), these data suggest that TGF-β1 and fortilin ELISA’s were capable of specifically detecting TGF-β1 and fortilin in Sera^TGF-β1+, fortilin+^, respectively.

We then subjected these sera to the modified ELISA using α-TGF-β1 capturing Ab and α-fortilin detecting Ab (Fig. 1e, upper panel). We found that Sera^TGF-β1+, fortilin+^ yielded ~10 times more signals than did Sera^TGF-β1-, fortilin-^ (Sera^TGF-β1+, fortilin+^ vs. Sera^TGF-β1-, fortilin-^ = 0.77 ± 0.049 vs. 0.08 ± 0.01 Abs_450_) (Fig. 1e, columns 1 vs. 2), showing that TGF-β1 captured by α-TGF-β1 capturing Ab was bound to fortilin, which was in turn detected by α-fortilin detecting Ab. These data suggest that fortilin and TGF-β1 form a complex in vivo in normal human sera.

Finally, we tested if fortilin could be co-purified by affinity column purification of TGF-β1 (Fig. S1b). After packing and equilibrating a gravity chromatography column with agarose beads conjugated to α-TGF-β1 antibody (Santa Cruz Biotechnology, Dallas TX, USA) (Figs. S1b, 1), we loaded it with Sera^TGF-β1+, fortilin+^ (Figs. S1b, 2), extensively washed it with Wash Buffer (phosphate-buffered saline (PBS)) (Figs. S1b, 3), and eluted the bound proteins by adding Elution Buffer (0.1 M Glycine HCl) (Figs. S1b, 4). We subjected the input (Sera^TGF-β1+, fortilin+^, Fig. S1c, INPUT), flow-through (Fig. S1c, FT), wash flow-throughs (Fig. S1c, WASH-1, -2, -4, and -5), and eluate (Fig. S1c, ELUATE) to western blot analysis using α-TGF-β1 and α-fortilin Abs. Strikingly, we found that the eluant contained both fortilin and TGF-β1 (Fig. S1c, ELUATE; Fig. S8), suggesting that affinity-purified TGF-β1 was bound to fortilin and that fortilin and TGF-β1 form a complex in vivo in normal human sera.

### Bio-layer interferometry (BLI) showed that fortilin binds TGF-β1

**Fig. 2:**
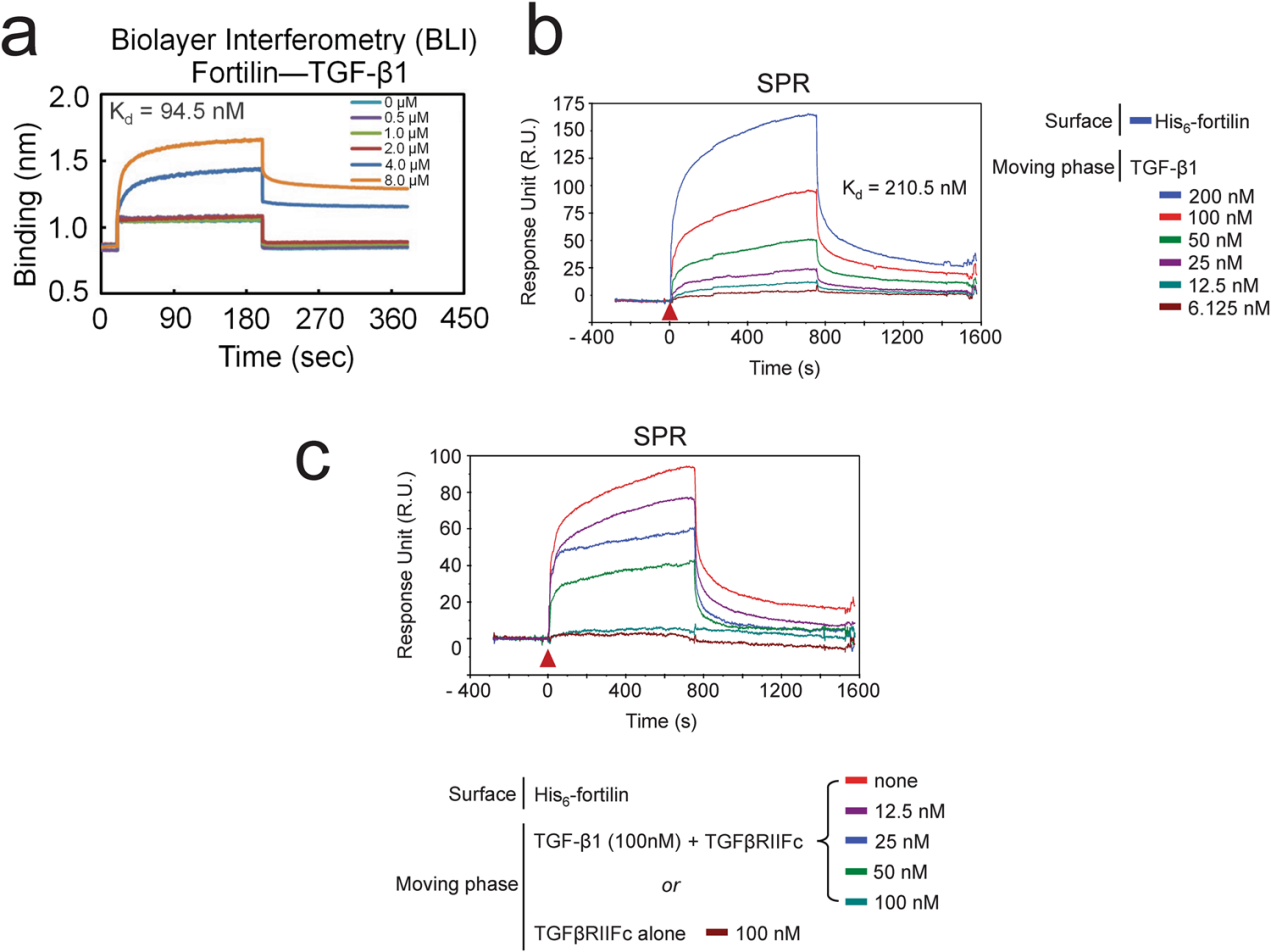
Both BLI and SPR show the specific interaction between fortilin and TGF-β1. BLI, bio-layer interferometry; K_d_, dissociation constant; SPR, surface plasmon resonance; His_6_-fortilin, hexa-his-tagged recombinant fortilin; TGFβRIIFc, the ligand-binding segment of the TGF-β receptor II conjugated to the Fc portion of the IgG. **a** BLI showed the specific binding of fortilin to TGF-β1 at K_d_ of 94.5 nM. **b** SPR showed that fortilin binds TGF-β1 at K_d_ of 210.5 nM. **c** After fortilin was conjugated to the SPR chip surface, TGFβRIIFc mixed with various concentrations of TGF-β1 was injected onto the chip surface to evaluate the binding of fortilin to TGF-β1 in the presence of TGFβRII. There was dose-dependent disruption by TGFβRII of the fortilin-TGF-β1 interaction. Because fortilin did not bind TGFβRIIFc (Fig. S2b), the data suggest that fortilin and TGFβRII competitively bind TGF-β1.

The above Co-IP, ELISA, and column experiments suggested that fortilin specifically interacts with TGF-β1 (Figs. 1, S1c). Next, we investigated binding between the two proteins using both BLI and surface plasmon resonance (SPR). For BLI assays, biotinylated rh-fortilin was immobilized on streptavidin-coated biosensors (ForteBio, Menlo Park, CA) and washed in PBS for 30 s. Various concentrations of rh-TGF-β1 were applied to the biosensors for 180 s to evaluate the association between the two proteins before rh-TGF-β1 solution was replaced by PBS for 180 s to evaluate their dissociation. Analyses of the binding data sets conducted using Blitz analysis software (Forte Bio) revealed an equilibrium dissociation constant (K_d_) of 94.5 nM, suggesting that both proteins interacted with each other specifically and at a medium intensity (Fig. 2a). TGF-β1 is synthesized as a precursor molecule comprising a signal peptide, LAP, and a mature TGF-β1 polypeptide^18^. Using the same methods, we tested if rh-fortilin interacted with recombinant human latency-associated-peptide (LAP)-TGF-β1 molecule. We found that the binding of fortilin to the LAP-TGF-β1 protein was weaker than that of fortilin to TGF-β1, at 0.93 µM (Fig. S1d).

### SPR showed that fortilin binds TGF-β1

For SPR assays, we cross-linked rh-fortilin on an SPR sensor chip and injected rh-TGF-β1 at increasing concentrations (0 to 200 nM) (Figs. 2b, S2a). We detected specific binding between the two proteins regardless of the location and nature of epitope tags, as indicated by the concentration-dependent increase in response units (Figs. 2b, S2a). To obtain the dissociation constant at equilibrium, we fitted the SPR response at steady state of TGF-β1 binding to N-terminal (Fig. 2b) His-tagged fortilin using the one site total binding model as implemented in GraphPad. We calculated a K_d_ of 210 nM, a value that is consistent with a medium affinity protein–protein interaction and similar to that from the BLI assay.

To gain greater molecular insights into the fortilin-TGF-β1 interaction, we used an SPR-based co-binding/inhibition assay that enables coarse epitope mapping (Fig. 2c)^19^. In this assay, a constant concentration of analyte (TGF-β1) was pre-incubated with increasing concentrations of the ligand-binding domain of the TGF-β1 receptor TGFβRII fused to IgG1 Fc (TGFβRII-Fc), a known interacting partner of TGF-β1. These TGFβ1–TGFβRII-Fc mixtures were injected onto a chip that had been cross-linked with fortilin. If TGFβRII-Fc and fortilin interact with the same epitope on TGF-β1, then we expected that higher TGFβRII-Fc concentrations would reduce the TGF-β1-dependent SPR signal. However, if TGFβRII-Fc and fortilin interact with different TGF-β1 surfaces, then we expected that higher TGFβRII-Fc concentrations would lead to an increased TGF-β1 SPR signal. Importantly, we found that TGFβRII-Fc reduced the TGF-β1 signal in a concentration-dependent manner, indicating that TGFβRII-Fc prevented binding of TGF-β1 to fortilin (Fig. 2c). IgG Fc alone (Fc) did not prevent TGF-β1 from binding fortilin (Fig. S2b, TGFβ1 + Fc), and Fc did not bind fortilin (Fig. S2b, Fc alone). These data are consistent with a model in which fortilin and TGFβRII contact the same surface on TGF-β1, thus providing direct evidence for a mechanism of TGF-β1 inhibition by fortilin.

### Fortilin prevents TGF-β1 from activating the Smad-binding element (SBE) in the cell

**Fig. 3:**
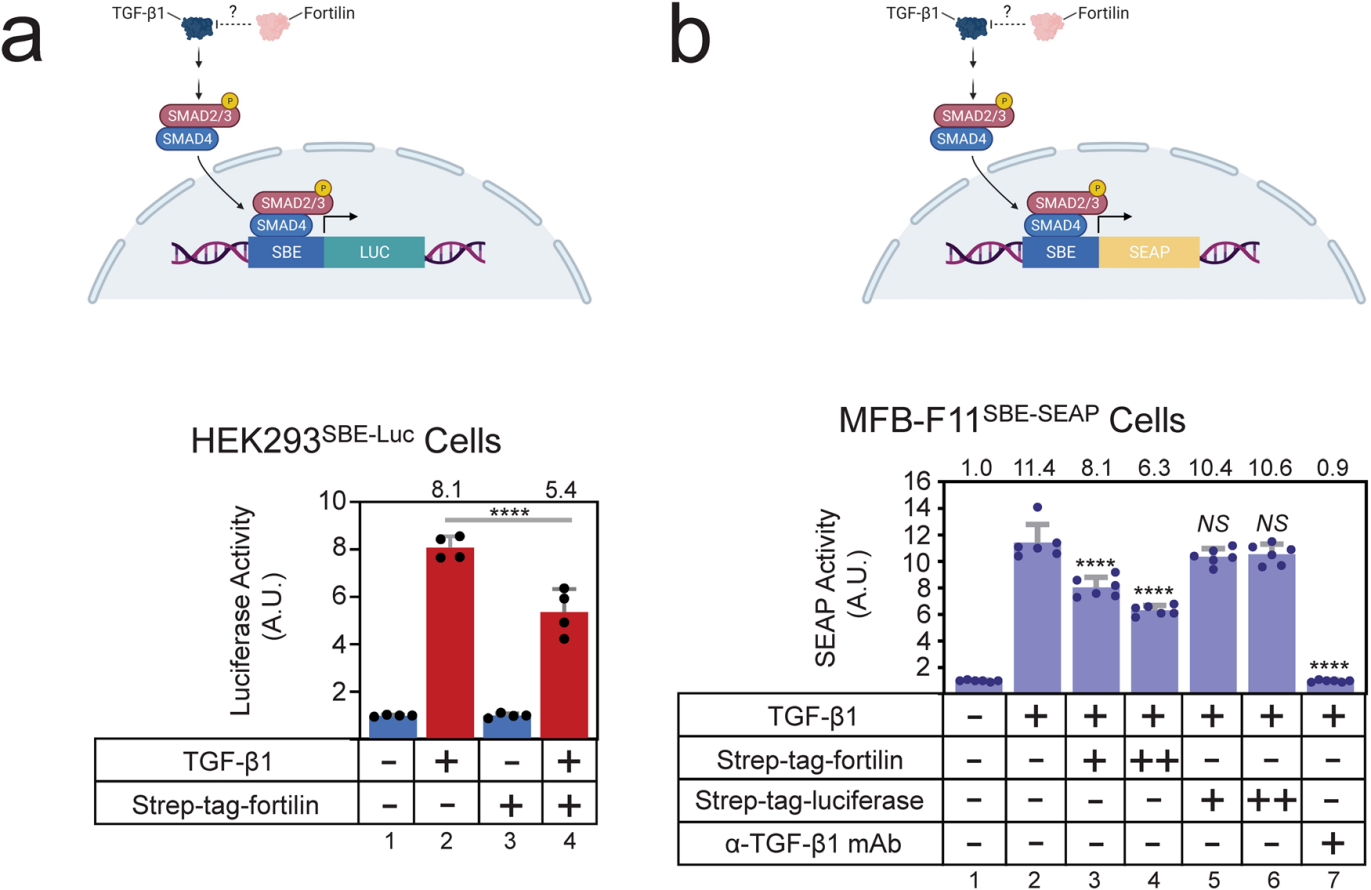
The luciferase assay shows that fortilin blocks TGF-β1-induced activation of the Smad2/3 binding element. strep-tag-fortilin, recombinant fortilin with strep-tag at its N-terminus; strep-tag-luciferase, recombinant luciferase with strep-tag at its N-terminus, used as the control; α-TGF-β1 mAb, neutralizing anti-TGF-β1 monoclonal antibody (1.25 µg/mL or 8.33 nM); HEK, human embryonic kidney cells; SBE-Luc, a vector containing the Smad2/3 binding element fused to the luciferase cDNA; A.U., arbitrary unit; SBE-SEAP, a vector containing the Smad2/3 binding element fused to the secreted embryonic alkaline phosphatase cDNA; MFB-F11^SBE-SEAP^ cells, immortalized mouse embryonic fibroblasts from *Tgfb1*^*−/−*^ mice that stably harbor the SBE-SEAP construct; data points, means ± SD; statistical analyses performed using ANOVA with Fisher’s multiple comparison; *N*, the number of biological replicates; NS, not statistically significant; ****P < 0.001. **a** Fortilin prevented TGF-β1 from activating the SBE in HEK293^SBE-Luc^ cells; 1 nM TGF-β1 and 1 nM strep-tag-fortilin were used. *N* = 4. **b** Fortilin, but not luciferase control protein, dose-dependently blocked TGF-β1-induced SBE activation in the MFB-F11^SBE-SEAP^ cells; 156 pM TGF-β1 was used to stimulate the cells. Moreover, 19.5 (low dose, +) and 195 (high dose, ++) nM strep-tag-fortilin or strep-tag-luciferase were used to block TGF-β1-induced SBE activation. *N* = 6.

The above data (Figs. 1, 2), when taken together, suggested that fortilin prevents TGF-β1 from ligating and activating TGFβRII, but we still did not know if this physical interaction has biological and functional significance. To test whether fortilin prevents TGF-β1 from activating the TGF-β1-Smad pathway in the cell, we first expressed strep-tagged fortilin (strep-tag-fortilin) and luciferase (strep-tag-luciferase) using 293T cells, purified them in the sterile condition, characterized them, and found that they were of appropriate sizes and acceptable purity without degradation (Fig. S3a; Figs. S9, S10). We then transiently transfected HEK293 cells with both the SBE-luciferase plasmid and the Renilla luciferase plasmid (the latter to evaluate transfection efficiency), treated them with TGF-β1 (1 nM) in the presence and absence of strep-tag-fortilin (3 nM), and subjected the cells to the dual luciferase assay as described in the Methods. Fortilin significantly decreased the TGF-β1-induced activation of SBE (luciferase activities; fortilin (−) vs. fortilin (+) = lanes 2 vs. 4 = 8.1 ± 0.2 vs. 5.4 ± 0.5 arbitrary units (A.U.), *P* < 0.001, *N* = 4 each) (Fig. 3a).

Next, we used the MFB-F11 cell line to validate these findings using a different cell system. The cell line was originally generated by stably transfecting mouse embryonic fibroblasts from *Tgfb1*^*−/−*^ mice with a construct in which the 12 CAGA boxes—Smad3/Smad4 binding sequences present in the human plasminogen activator inhibitor-1 gene^20^—are fused to a secreted alkaline phosphatase (SEAP) reporter gene^21,22^. This cell line has been shown to respond to TGF-β1 in a linear fashion from 1 pg/mL to 10 ng/mL (0.04–400 pM), which is an extremely wide dynamic range (ref. ^21^ and Fig. S3b). We first stimulated the MFB-F11 cells with 156 pM (2 ng/mL) of recombinant TGF-β1 and found that the SEAP activity increased 11.4-fold (Fig. 3b, lanes 1 vs. 2). Addition of strep-tag-fortilin (Fig. 3b, lanes 3 and 4), but not strep-tag-luciferase (Fig. 3b, lanes 5 and 6), prevented TGF-β1 from activating its signaling pathway in a dose-dependent fashion (Fig. 3b, lanes 2 vs. 3 vs. 4 = 11.4, 8.1, and 6.3 A.U. for 0, 19.5, and 195 nM fortilin, respectively, *P* < 0.001 by one-way analysis of variance (ANOVA), Fisher pairwise comparisons). These data suggest that the functional significance of fortilin binding to TGF-β1 is the inhibition of the TGF-β1 signaling pathway.

### Fortilin prevents TGF-β1 from phosphorylating Smad3

**Fig. 4:**
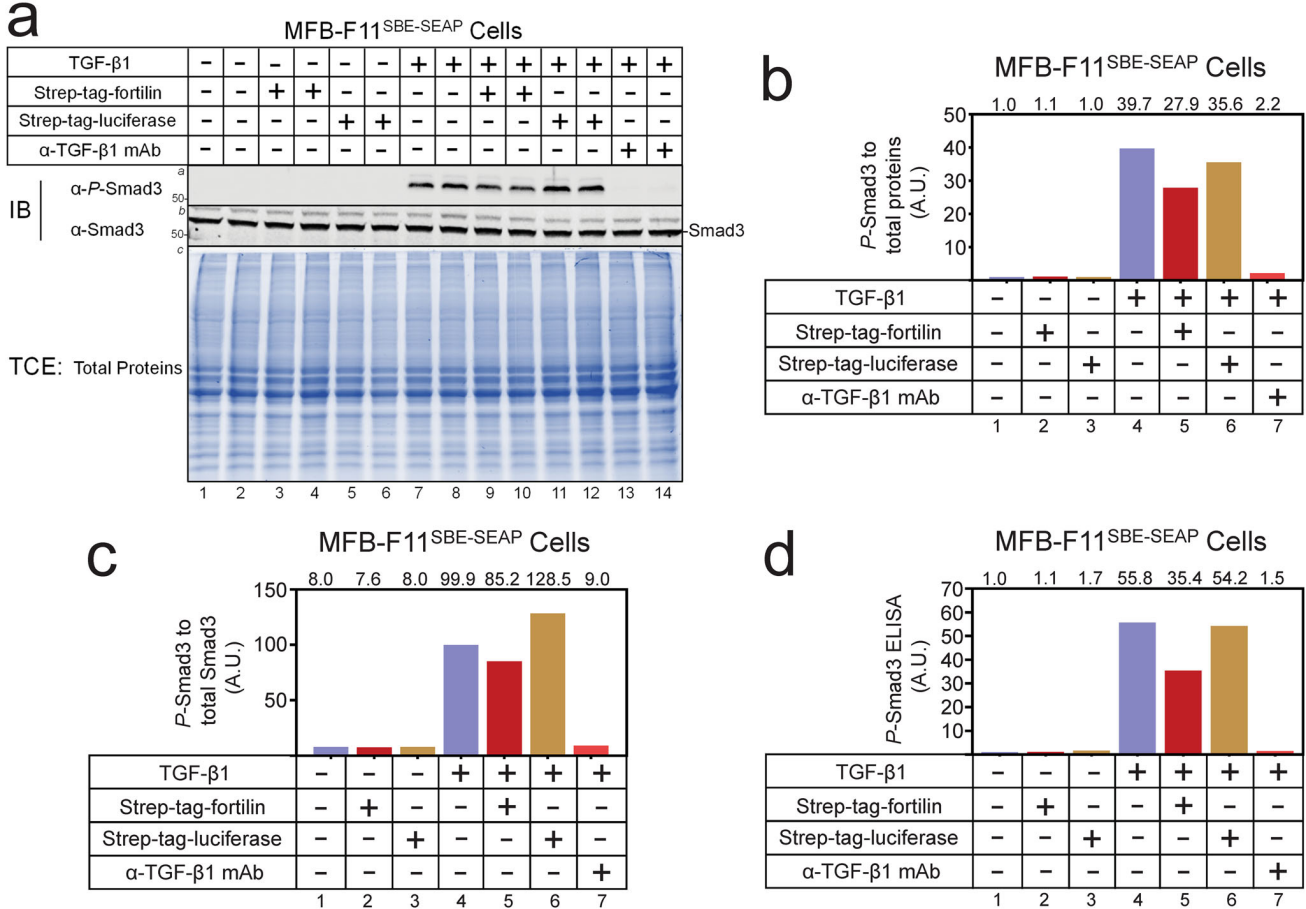
Quantitative western blots and ELISA show that fortilin blocks TGF-β1-induced phosphorylation of Smad3. SBE-SEAP, a vector containing the Smad2/3 binding element fused to the secreted embryonic alkaline phosphatase cDNA; MFB-F11^SBE-SEAP^ cells, immortalized mouse embryonic fibroblasts from *Tgfb1*^*−/−*^ mice that stably harbor the SBE-SEAP construct; strep-tag-fortilin, recombinant fortilin with strep-tag at its N-terminus (19.5 nM); strep-tag-luciferase, recombinant luciferase with strep-tag at its N-terminus, used as the control (19.5 nM); α-TGF-β1 mAb, neutralizing α-TGF-β1 monoclonal antibody (1.25 µg/mL or 8.33 nM); IB, immunoblot; α-*P*-Smad3, anti-phosphorylated Smad3 antibody; α-Smad3, anti-Smad3 antibody; TCE, 2,2,2-trichloroethanol; A.U., arbitrary unit. **a** Western blot analysis using α-*P*-Smad3 and α-Smad3 and total protein visualization by TCE. **b**, **c** Quantification of *P*-Smad3, normalized to total proteins (**b**) and total Smad3 (**c**), showing that fortilin, but not luciferase control protein, prevented TGF-β1 from phosphorylating Smad3. **d** ELISA of *P*-Smad3 on the lysates from MFB-F11^SBE-SEAP^ cells also showed that fortilin, but not luciferase control protein, prevented TGF-β1 from phosphorylating Smad3.

To further validate that fortilin prevents TGF-β1 from activating the TGF-β1 pathway, we evaluated the status of Smad3 phosphorylation (*P*-Smad3) in TGF-β1-stimulated MFB-F11^SBE-SEAP^ cells in the presence and absence of fortilin, using quantitative western blot analysis (Fig. 4a–c; Fig. S7), where *P*-Smad3 signals were normalized to either total proteins loaded (Fig. 4b) or total Smad3 (Fig. 4c). In the absence of TGF-β1, neither recombinant fortilin (strep-tag-fortilin) nor its control recombinant luciferase (strep-tag-luciferase) induced Smad3 phosphorylation (Fig. 4a, lanes 1–6; Fig. 4b, lanes 1–3; Fig. 4c, lanes 1–3). TGF-β1, but not TGF-β1 in the presence of anti-TGF-β1 antibody, induced the phosphorylation of Smad3 (Fig. 4a, lanes 7 & 8 vs. 13 & 14; Fig. 4b, lanes 4 vs. 7; Fig. 4c, lanes 4 vs. 7). In this system, recombinant fortilin, but not control recombinant luciferase, decreased the TGF-β1-induced phosphorylation of Smad3 (Fig. 4a, lanes 9 & 10 vs. 11 & 12; Fig. 4b, lanes 5 vs. 6; Fig. 4c, lanes 5 vs. 6). To further validate this finding, we subjected the cell lysates from the same experiment to ELISA of *P*-Smad3. Consistently, the data showed that TGF-β1-induced phosphorylation of Smad3 was inhibited by recombinant fortilin (Fig. 4d, lanes 4 vs. 5) but not by recombinant luciferase (Fig. 4d, lanes 4 vs. 6).

### Fortilin inhibits TGF-β1-induced smooth muscle marker expression in C3H10T1/2 cells

**Fig. 5:**
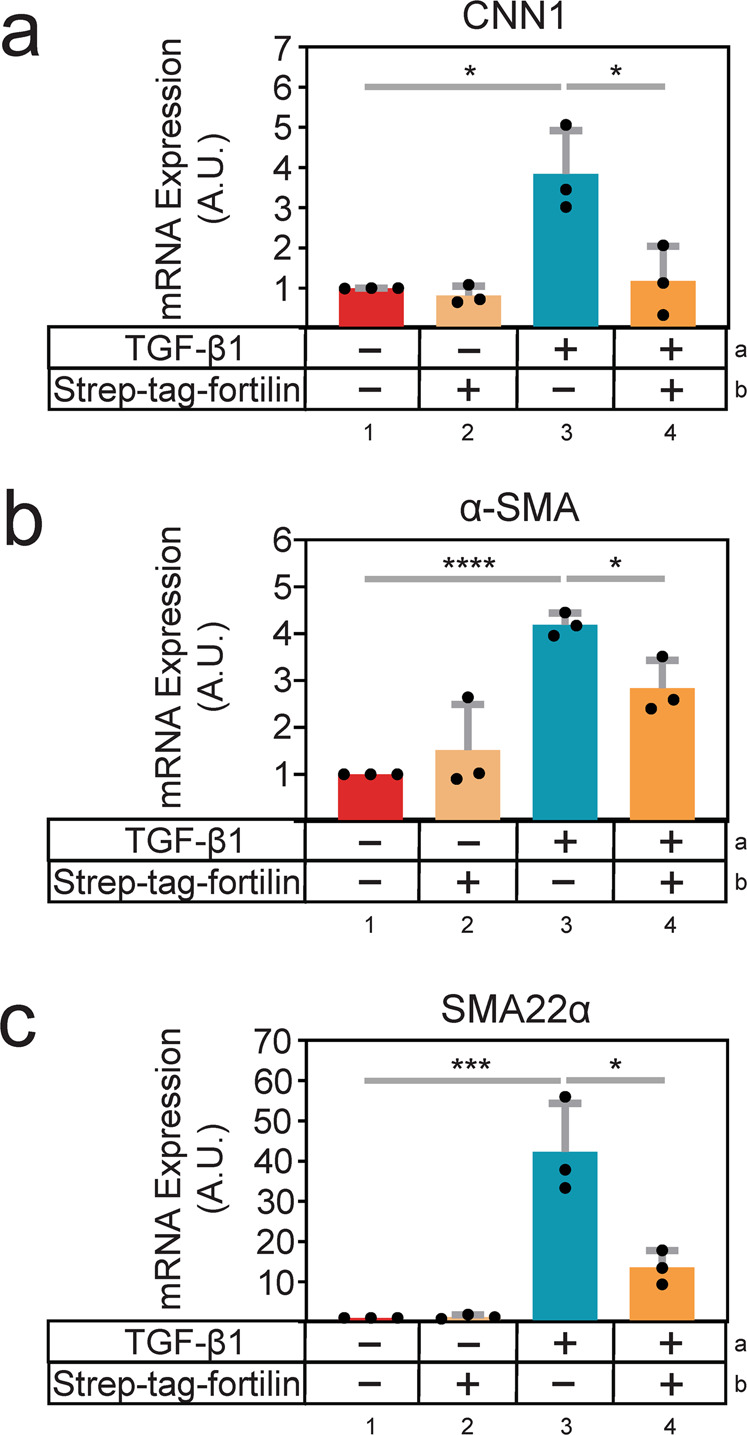
Fortilin inhibits TGF-β1-induced differentiation of the C3H10T1/2 mesenchymal progenitor cells. CNN1, Calponin 1; α-SMA, alpha-smooth muscle actin; SMA22α, smooth muscle protein 22α; A.U. arbitrary unit; Data points, means ± SD; *N*, the number of biological replicates; *P < 0.05, ***P < 0.005; ****P < 0.001. C3H10T1/2 cells were starved with DMEM containing 0.1% fetal bovine serum for 48 h followed by incubation with fortilin (0.5 µg/mL), TGF-β1 (1 ng/mL), or their mixture for 8 h. Untreated cells were used as the negative control. Quantitative real-time PCR was performed (*N* = 3) to determine mRNA expression of CNN1 (**a**), α-SMA (**b**), and SMA22α (**c**) in C3H10T1/2 cells. CYP was used the internal reference. Fortilin inhibited TGF-β1-induced expression of the smooth muscle cell differentiation marker proteins (CNN1, α-SMA, and SMA22α) in C3H10T1/2 cells.

In atherosclerosis, smooth muscle cells (SMCs) accumulate in the intimal space, produce extracellular matrix (ECM), and contribute to the expansion of the plaque^23^. There are several theories about the source of intimal SMCs: (i) dedifferentiated medial SMC, (ii) adventitial progenitor cells, (iii) medial progenitor cells, or (iv) bone marrow progenitor cells^23^. TGF-β1 has been shown to play an important role in both (i) differentiation of progenitor cells to SMCs and (ii) maturation of proliferative SMCs to contractile and quiescent SMCs^23^. To investigate the role of fortilin in TGF-β1-induced differentiation of progenitor cells to SMCs, we evaluated the mRNA expression of SMC differentiation markers CNN1 (Fig. 5a)^24^, α-SMA (Fig. 5b)^25^, and SMA22α (Fig. 5c)^25^ in C3H10T1/2 cells exposed to TGF-β1, fortilin, or their mixture. The C3H10T1/2 cell line, originally established from C3H mouse embryos^26^, displays attribute similar to those of mesenchymal stem cells and, when treated with TGF-β1, cells differentiate to become vascular SMCs^27^. C3H10T1/2 cells express no epithelial markers without stimulation^28^. Stimulation by TGF-β1 (Fig. 5a–c, lanes 1 vs. 3), but not by fortilin (Fig. 5a–c, lanes 1 vs. 2), dramatically enhanced mRNA expression of the differentiation markers as determined by quantitative real-time PCR assays. Strikingly, recombinant fortilin (strep-tag-fortilin) abrogated TGF-β1-induced upregulation of the SMC differentiation markers compared with the control (Fig. 5a–c, TGF-β1 (lane 3) vs. TGF-β1 + fortilin (lane 4)). These data suggest that fortilin prevents TGF-β1 from differentiating C3H10T1/2 progenitor cells to SMCs and that fortilin is an inhibitor of TGF-β1.

### Structural basis for inhibition of the TGF-β1 pathway by fortilin

**Fig. 6:**
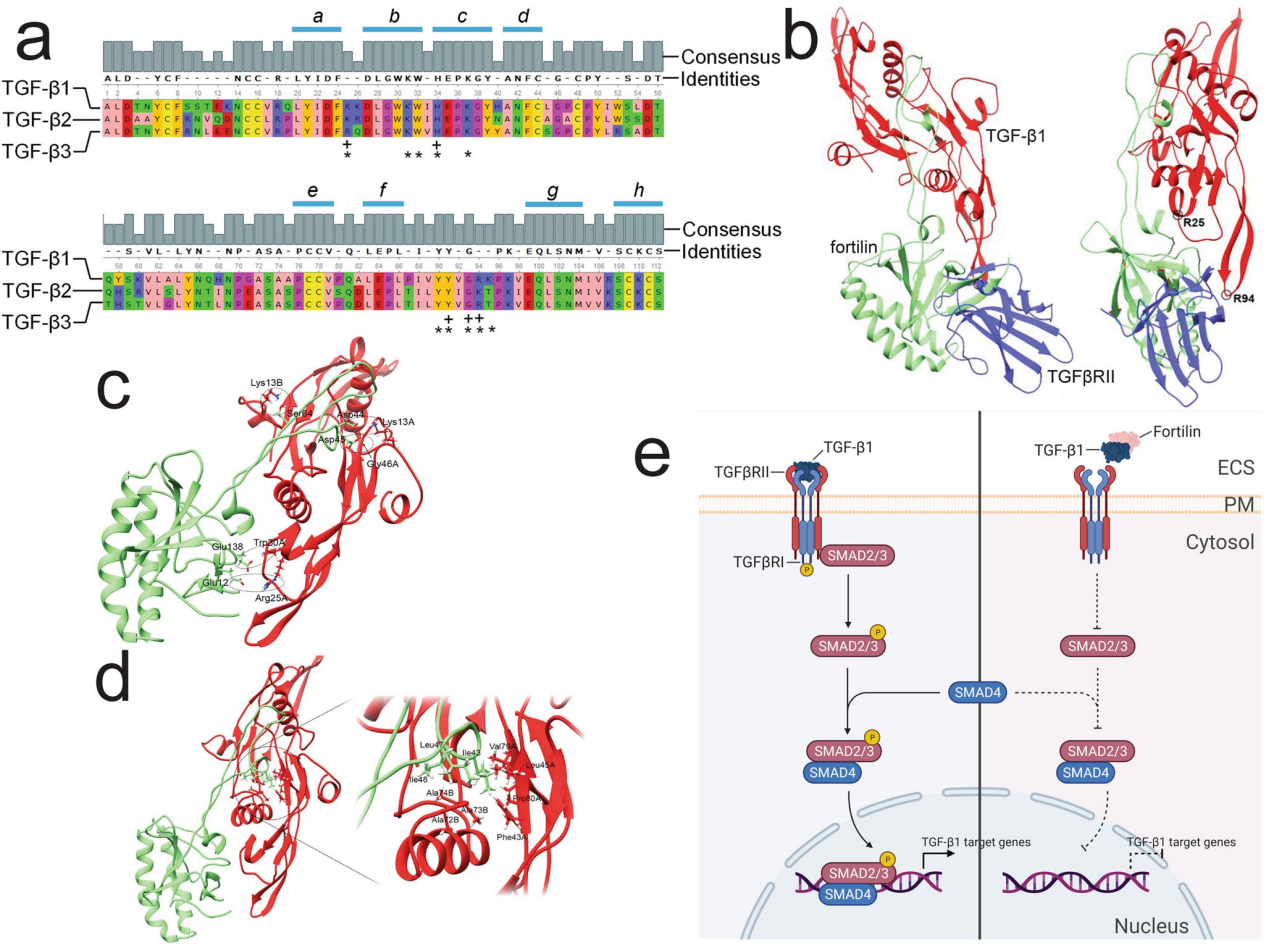
Fortilin occupies the binding space for TGF1-β1 that is normally occupied by TGFβRII. +, amino acid residues interfacing between TGF-β1 and TGFBRII; *, amino acid residues interfacing between TGF-β3 and TGFBRII; Identities, the amino acid residues of TGF-β2 and TGF-β3 that are identical to those of TGF-β1; ECS, extracellular space; PM, plasma membrane; P, phosphorylated amino acid residue; TGFβRI, TGF-β1 receptor I; TGFβRII, TGF-β1 receptor II. **a** Sequence alignment of TGF-β1, -β2, and -β3. Five amino acids (Arg^25^, His^34^, Tyr^91^, Gly^93^, and Arg^94^) of TGF-β1 (+) and 10 amino acids (Arg^25^, Lys^31^, Trp^32^, His^34^, Lys^37^, Tyr^90^, Tyr^91^, Gly^93^, Arg^94^, and Thr^95^) of TGF-β3 (*) interface with TGFβRII according to the prior co-crystallization studies. **b** Computational modeling of dimerized TGF-β1 and either fortilin or TGFβRII. Fortilin (green) and TGFβRII (blue) occupy the same spatial location in relation to dimerized TGF-β1 (red, chains A and B) and either fortilin or TGFβRII, but not both, can bind dimerized TGF-β1 at a given time. Fortilin and TGFβRII are depicted in an overlaid fashion. The small open circles denote arginine (Arg) residues. **c** Structural basis of the fortilin-TGF-β1 interaction—hydrogen bonding. Glu^12^, Glu^138^, Asp^44^, and Asp^45^ of fortilin create hydrogen bonding with Arg^25^, Trp^30^, Lys^13^, and Gly^46^ of one of the TGF-β1 molecules (chain A). **d** Structural basis of the fortilin-TGF-β1 interaction—hydrophobic binding surface. The hydrophobic region of the binding interface is formed by (a) Ile^43^, Leu^47^, and Ile^48^ of fortilin, (b) Phe^43^, Leu^45^, Val^79^, and Pro^80^ of the first TGF-β1 molecule (chain A), and (c) Ala^72^, Ala^73^, and Ala^74^ of the second TGF-β1 molecule (chain B). **e** The role of fortilin in the regulation of the TGF-β1 pathway. TGFβRII, when ligated by TGF-β1, recruits and phosphorylates TGFβRI, which in turn phosphorylates Smad2/3. Phosphorylated Smad2/3 enters the nucleus and activates TGF-β1 target genes (left panel). Fortilin binds TGF-β1 and prevents TGF-β1 from ligating and activating TGF-β1 receptors (right panel).

Because it interacts with TGF-β1, TGF-β2, and TGF-β3 (Fig. 1), fortilin is likely to bind the conserved segments of these three isoforms. We first aligned the isoforms and found that TGF-β1 and TGF-β2 are 71% identical, TGF-β1 and TGF-β3 are 77% identical, and TGF-β2 and TGF-β3 are 79% identical. There are eight highly conserved segments among the three proteins (Fig. 6a, segments *a–h﻿*). In addition, Radaev et al. reported co-crystallization of TGF-β1 and TGFβRII (Protein Data Base [PDB] ID: 3KFD) and found that the interaction between these two proteins involves the following five TGF-β1 residues: Arg^25^, His^34^, Tyr^91^, Gly^93^, and Arg^94^ (Fig. 6a, +)^29^. They also noted that the interaction between TGF-β2 and TGFβRII is much weaker than those between TGF-β1/3 and TGFβRII based on SPR analyses^29^. Hart et al. performed co-crystallization of TGF-β3 and TGFβRII (PDB: 1KTZ) and identified 10 amino acids residues located at the TGF-β3-TGRβRII interface: Arg^25^, Lys^31^, Trp^32^, His^34^, Lys^37^, Tyr^90^, Tyr^91^, Gly^93^, Arg^94^, and Thr^95^ (Fig. 6a, *)^30^. Because five amino acids (Arg^25^, His^34^, Tyr^91^, Gly^93^, and Arg^94^) of TGF-β1/3 are common between the TGF-β1-TGFβRII and TGF-β3-TGFβRII interfaces and because our SPR data suggested that fortilin binds TGF-β1 through the TGF-β1-TGFβRII interface (Fig. 2c), it is likely that fortilin interacts with TGF-β1 through one of these five amino acids. Arg^25^ and His^34^ of TGF-β1/3 within the highly conserved segments a–c represent the most promising amino acids participating in the interaction with fortilin (Fig. 6a).

To evaluate how fortilin and TGFβRII structurally compete for TGF-β1, we performed computational modeling of dimerized TGF-β1 (PDB ID: 3KFD) and either fortilin (PBD ID: 2HR9) or TGFβRII (PDB ID: 5TY4) in ClusPro^31–34^. We found that fortilin and TGFβRII occupy the same spatial location in relation to dimerized TGF-β1, suggesting that either fortilin or TGFβRII, but not both, can bind dimerized TGF-β1 at a given time (Fig. 6b, blue, TGFβRII; red, TGF-β1 dimer; green, fortilin). We then evaluated the nature of the molecular interaction between the fortilin and TGF-β1 dimer. We found that Glu^12^, Glu^138^, Asp^44^, and Asp^45^ of fortilin created hydrogen bonding with Arg^25^, Trp^30^, Lys^13^, and Gly^46^ of one of the TGF-β1 molecules (A), respectively (Fig. 6c). Intriguingly, Arg^25^ of TGF-β1/3 has been shown to interface with TGFβRII in crystallographic studies^29,30^ (Fig. 6a, * & +). We also found that the hydrophobic region of the binding interface was formed by (a) Ile^43^, Leu^47^, and Ile^48^ of fortilin; (b) Phe^43^, Leu^45^, Val^79^, and Pro^80^ of the first TGF-β1 molecule (A); and (c) Ala^72^, Ala^73^, and Ala^74^ of the second TGF-β1 molecule (B) (Fig. 6d), suggesting that the thermodynamic stability of the fortilin-TGF-β1 interaction is attained by both hydrogen bonding and hydrophobic interaction between the two molecules.

In conclusion, we showed that fortilin specifically interacts with TGF-β1 using Co-IP-western blot analysis (Fig. 1a), the modified ELISA system (Fig. 1e), the column affinity co-purification assays (Figs. S1b, c), BLI analysis (Fig. 2a), and SPR assays (Fig. 2b). The binding of fortilin to TGF-β1 prevented it from ligating TGFβRII as shown by SPR (Fig. 2c), inhibited TGF-β1-induced activation of the SBE in both luciferase assays in HEK293 cells (Fig. 3a) and SEAP assays in MFB-F11 cells (Fig. 3b), and blocked the phosphorylation of Smad3 as determined by both western blot analysis (Fig. 4a–c) and ELISA (Fig. 4d). Fortilin also prevented TGF-β1 from differentiating C3H10T1/2 mesenchymal pluripotent cells into SMCs (Fig. 5). When taken together with prior crystallographic studies, the computational docking study results (Fig. 6b–d) and analyses of TGF-β amino acid sequences (Fig. 6a) also support the fortilin-TGF-β1 interaction. Our results suggest that fortilin specifically binds TGF-β1 and prevents it from ligating and activating TGFβRII (Fig. 6e), although further investigation is necessary to determine the role of the fortilin-TGF-β1 interaction in human diseases.

## Discussion

The most substantial findings of this study are the specific physical interaction between fortilin and TGF-β1 and elucidation of the biological significance of this interaction, namely that fortilin negatively regulates the ability of TGF-β1 to activate its canonical pathway through TGFβRII.

Because the serum concentration of TGF-β1 is drastically higher than those of TGF-β2 and TGF-β3 (Fig. S1a), we focused on the interaction between fortilin and TGF-β1 in the current work. Although the fortilin-TGF-β1 interaction may represent the majority of fortilin-TGF-β interactions in the blood, further investigation is called for to characterize and elucidate the biological significance of the fortilin-TGF-β2/3 interaction.

Although fortilin specifically binds TGF-β1, the fortilin-TGF-β1 interaction is weaker than the interaction between TGF-β1 and TGFβRII. As we previously reported, the K_d_ between TGF-β1 and TGFβRII is 1.49 nM^35^, whereas the K_d_ of the TGF-β1-fortilin interaction is 94.5–210.5 nM (Fig. 2a, b). This suggests that once TGF-β1 binds TGFβRII, fortilin is not capable of disrupting the ligation, which is also consistent with the dose-dependent disruption by TGFβRII of the fortilin-TGF-β1 interaction shown in Fig. 2c. Fortilin may interfere with the biological activities of TGF-β1 by binding and trapping it before it reaches TGFβRII, its receptor.

It has been established that TGF-β binds directly to TGFβRII, which is a constitutional kinase. Bound TGF-β is then recognized by TGFβRI, which is recruited to the TGF-β-TGFβRII complex and becomes phosphorylated and activated by TGFβRII. Activated TGFβRI in turn phosphorylates and activates Smad3. In other words, Smad3 is phosphorylated only when TGFβRI is recruited to the TGF-β-TGFβRII complex and activated by phosphorylation^36–39^. Our data showing that fortilin inhibits TGF-β1-induced Smad3 phosphorylation (Fig. 4) suggest that fortilin inhibits the recruitment of TGFβRI to TGFβRII by preventing TGF-β1 from ligating TGFβRII but not by preventing the formed TGF-β1-TGFβRII complex from recruiting TGFβR1, as the TGF-β1-TGFβRII interaction is stronger than the TGF-β1-fortilin interaction (1.49 nM^35^ versus 94.5–210.5 nM, Fig. 2a, b).

TGF-β1 also interacts with many molecules other than fortilin, as reported in the literature. These TGF-β1-interacting proteins are localized in (a) the blood—thrombospondin-1 (THBS1)^40–42^, α2-macroglobulin (A2M)^40–42^, pregnancy zone protein (PZP)^43^, β-amyloid^1-40^^43^, and follistatin-like-1 (FSTL1)^43^; (b) the extracellular matrix—LAP^18,44^, asporin^45^, decorin^46^, fibrinogen^47^, fibrinogen β-chain (FGB)^48^, biglycan^49^, fibromodulin^49^, lumican^49^, collagen type 1α1 (COL1A1)^50^, and collagen type 2α1 (COL2A1)^51^; and (c) the plasma membrane—endoglin^52–56^, activin A receptor-like type 1^57,58^, and FSTL1^59,60^.

These TGF-β1-interacting proteins have been demonstrated to have diverse biological impacts on TGF-β1 signaling. They can be either positive, as with THBS1^40–42^, fibrinogen^47^, FGB^48^, endoglin^52–56^, activin A receptor-like type 1^58^, and FSTL1^59,60^ or (b) negative, as with asporin^45^, decorin^46^, A2M^61^, and PZP^43^. On the other hand, TGF-β1 has been shown to impact the biological activity of TGF-β1-binding proteins either positively or negatively. For example, TGF-β1 directly binds the 40-residue Aβ peptide (Aβ^1-40^), facilitates the oligomerization of Aβ^1-40^, and enhances Aβ^1-40^-mediated toxicity in PC12 cells^62^. TGF-β1 binds the 50–55 kDa secreted protein FSTL1 and prevents it from interacting with its putative transmembrane receptor (disco-interacting protein 2)^59^. The biological significance of the TGF-β1 interaction with biglycan^49^, fibromodulin^49^, lumican^49^, COL1A1^50^, and COL2A1^51^ remains unknown.

Fortilin has no sequence similarity to any of the above TGF-β1-interacting proteins, and it possesses several unique attributes that they do not have. First, fortilin is present not only in the extracellular space where it interacts with TGF-β1 but also in the nucleus, ER, and cytosol where it interacts with p53^12^, IRE1α^5^, and MCL1^15^, respectively. Second, fortilin is a multi-functional protein: in addition to binding and inhibiting TGF-β1, fortilin protects cells against apoptosis by (i) binding and inhibiting the pro-apoptotic and tumor suppressor protein p53^12^, (ii) binding and protecting against degradation of the anti-apoptotic Bcl-2 family member protein MCL1^63^, (iii) binding calcium (Ca^2+^) and preventing Ca^2+^ from activating Ca^2+^-dependent apoptosis pathway^64^, and (iv) binding and preventing IRE1α from activating the ER stress-induced apoptosis pathway^5^. Fortilin also binds and potentiates the antioxidant activity of peroxiredoxin-1^7^. Further, fortilin is required for normal cell cycle progression through the stabilization of microtubules^6^. Finally, we do not know if fortilin in the extracellular space has functions other than binding to and blocking the signal transduction of TGF-β1. We also do not know if TGF-β1 modulates the function of extracellular fortilin. Further investigation is needed to address these questions.

TGF-β1 is involved in complex and diverse biological activities in all organisms. TGF-β1 can suppress early-stage tumors through its potent antiproliferative activity and the induction of cell differentiation and apoptosis^65,66^. In advanced cancer, however, TGF-β1 actually promotes tumor invasion and metastasis by inducing the epithelial-mesenchymal transition, facilitating tumor angiogenesis, and hampering tumor immune surveillance by the host^65–67^. TGF-β1 is also implicated in many fibrotic human diseases, including pulmonary fibrosis^68^. Finally, TGF-β1 plays critical roles in the negative regulation of the inflammatory immune response^69^. The results of this study showing the physically and biologically meaningful interaction between fortilin and TGF-β1 represent the beginning of our long-term investigation of the regulatory role of fortilin in numerous TGF-β1-mediated biological processes and human diseases, including cancer invasion, metastasis, immune surveillance, and angiogenesis; pulmonary fibrosis and other fibrotic diseases; and inflammatory diseases. Although its activities appear to be expansive and complex, it is also possible that fortilin orchestrates seemingly diverse biological events to achieve a simple cellular goal in response to certain microenvironmental changes.

## Methods

### Reagents and materials

Recombinant human TGF-β1, -β2, and -β3 (rh-TGF-β1, -β2, β3) for Co-IP were obtained from eBioscience (San Diego, CA, USA; Catalog #: 14-8348-62), R&D Systems (Minneapolis, MN, USA; Catalog #: 302-B2-010/CF), and R&D Systems (Catalog #: 243-B3-010/CF), respectively. Recombinant human latency-associated-peptide (LAP)-TGF-β1 protein was purchased from R&D Systems (Catalog #: 246-LP-025/CF). Neutralizing anti-TGF-β antibody (Ab) was obtained from R&D Systems (Catalog #: MAB1835-100, Clone: 1D11). Recombinant mouse TGF-β1 for luciferase, SEAP reporter, and Smad3 phosphorylation assays on the MFB-F11 cells were purchased from R&D Systems (Catalog #: 7666-MB/CF). Recombinant human TGF-β1 for C3H10T1/2 cell differentiation assays was purchased from R&D Systems (Catalog #: 240-B-010). Human α-thrombin was purchased from Haematologic Technologies (Catalog #: HT-0020, Essex Junction, VT, USA). Agarose beads conjugated to anti-TGF-β1 monoclonal antibody (Clone: 3C11) were purchased from Santa Cruz Biotechnology (Dallas, TX, USA; Catalog #: sc-130348 AC).

### Cell culture

MycoFluor™ (Thermo Fisher Scientific-Molecular Probe, Eugene, OR, USA) was used to detect mycoplasma contamination when appropriate. HEK293 (ATCC® CRL-1573™) cells were obtained from American Type Culture Collection (ATCC, Manassas, VA, USA). Cells were maintained in Dulbecco’s modified Eagle’s medium (DMEM) (Corning, Corning, NY, USA; Catalog #: 0-013-CV) with 10% fetal bovine serum (FBS) (Gibco Thermo Fisher, Waltham, MA, USA; Catalog #: 10082-147) at 37 °C in an atmosphere containing 5% CO_2_. MFB-F11 cells^21,22^ were a kind gift from Dr. Tony Wyss-Coray (Stanford University, Stanford, CA, USA). The cells were generated at the Wyss-Coray lab by stably transfecting mouse embryonic fibroblasts from *Tgfb1*^*−/−*^ mice with a synthetic promoter element containing twelve CAGA boxes fused to a SEAP reporter gene^22^. The cells were initially propagated in DMEM supplemented with 10% FBS.

### Western blot analyses

SDS-PAGE and western blot analyses were performed as described previously^2,14,15,64,70^ on the lysates from the cell pellets. The following primary antibodies were used at the indicated dilutions/concentrations: Anti-fortilin (Abcam, Waltham, MA, USA; Clone EPR5540, ab133568; 1:2000 dilution) for Fig. S1c; anti-fortilin (MRB International, Woburn, MA, USA; Catalog #: PM017; 1:1000 dilution) for Fig. S3; anti-Gaussia luciferase (GLuc, NEB, Ipswich, MA, USA; Catalog #: E8023; 1:500 dilution); anti-TGF-β1 (Abcam; Clone EPR18163, ab179695; 1:1000 dilution) for Western blot analysis of Fig. 1a and Fig. S1c; anti-TGF-β1/2/3 (denoted anti-TGF-β) (R&D Systems, Minneapolis, MN, USA; Clone 1D11, MAB1835-100; 1:1000 dilution) for western blot analysis of Fig. 1b; anti-Flag (Sigma, St. Louis, MO, USA; Clone M2, F1804; 1:1000 dilution); anti-His_6_ (Abcam; Clone HIS.H8, ab18184; 1:1000 dilution); anti-GAPDH (Santa Cruz Biotechnology, Dallas, TX, USA; Clone 6C5, sc-32233; 1:10,000 dilution); anti-Strep-tag (IBA Lifesciences, Göttingen, Germany; 2-1507-001; 1:1000 dilution); anti-*P*-Smad3 (Rockland Immunochemicals, Limerick, PA, USA; Clone AF9F7, 600-401-919; 1:1000 dilution); anti-Smad3 (Abcam; Clone HIS.H8, ab75512; 1:1000 dilution). All antibodies were used with appropriate IRDye680LT- or IRDye800CW-conjugated secondary antibodies (LI-COR, Lincoln, NE, USA). Images were electronically captured using the Bio-Rad ChemiDoc MP Imaging System, and the signal intensities of protein bands were quantified using Image Lab Software (Bio-Rad, Hercules, CA, USA). To quantify *P*-Smad3 expression in relation to total proteins loaded, 0.5% (v/v) 2,2,2-trichloroethanol (TCE) was added to a polyacrylamide gel before polymerization. After standard SDS-PAGE, the gel was UV-irradiated on the Bio-Rad ChemiDoc MP Imaging System for 2 min, its image was electronically captured, and the cumulative band densities were calculated to assess loading conditions as previously described^71^. The signal intensity of western blot bands of *P*-Smad3 was then divided by that of the TCE bands to derive the pSmad3 expression index, which was expressed as A.U. For the quantification of *P*-Smad3 expression in relation to total Smad3, the signal intensity of *P*-Smad3 bands quantified by Image Lab Software (Bio-Rad) was divided by that of corresponding total Smad3 bands, which was expressed as A.U.

### Generation of recombinant human fortilin

Recombinant human fortilin (rh-fortilin) was purified using the Strep-tag purification system (IBA LifeSciences) as described previously^5^ with slight modification. Briefly, 293T cells stably expressing human fortilin with an N-terminal Strep-tag II tag (peptide sequence = WSHPQFEK) were collected, washed in PBS, and resuspended in Buffer W (100 mM Tris HCl, pH8, 150 mM NaCl, 1 mM EDTA, and protease inhibitor cocktail, 1 tablet/mL). The cell suspension then was lysed by repeated freeze-thaw cycles and sonicated to shear the genomic DNA. After centrifugation to remove the cell pellet, the cleared cell lysate was passed through a gravity flow Strep-Tactin® XT Superflow® high-capacity column (IBA LIfeSciences). Next, the column was washed five times with Buffer W and eluted with Buffer BXT (Buffer W containing 50 mM biotin). The Strep-tagged fortilin eluent fractions were pooled and concentrated using ultra centrifugal filter units (Amicon™, Millipore Sigma, Burlington, MA, USA). The concentrated proteins were dialyzed in PBS using a Slide-A-Lyzer™ MINI dialysis device (Thermo Fisher Scientific, Waltham, MA, USA). Finally, purified rh-fortilin was characterized by Stain-free total protein TCE® (Bio-Rad) and western blot analyses.

### Co-IP-western blot analysis

*Co-IP of TGF-β1 by fortilin*: To evaluate whether fortilin specifically interacts with TGF-β1, we added recombinant human (rh) flag-tagged fortilin (OriGene, Rockville, MD, USA; Catalog #: TP301664), rh-flag-tagged p53 (OriGene, Catalog #: TP300003), rh-His_6_-tagged NQO2 (Abcam, Catalog #: ab93933), and rh-TGF-β1 (eBioscience, Catalog #: 14-8348-62) to Bufffer A (10 mM Tris, pH 7.4, 100 mM NaCl, 0.01% NP-40, 2 mM phenylmethylsulfonyl fluoride (PMSF), protease inhibitor cocktail, 1 tablet/mL), mixed the solution by pipetting, took 10% of the total volume for the analysis of input, and added a 100 µL aliquot to Tube A and to Tube B. We added 3 µg each of rabbit IgG isotype control (Thermo Fisher Scientific, Catalog #: AB-105-C) and rabbit anti-fortilin antibody (Abcam, Catalog #: ab133568, Clone: EPR5540) to Tubes A and B, respectively, and incubated them at room temperature for 2 h. We then added 50 µL of pre-washed Dynabeads™ M-280 (Thermo Fisher Scientific) coated with sheep anti-rabbit IgG to these tubes to immunoprecipitate the immune complexes that had formed. We washed the beads four times using Buffer B (10 mM Tris, pH 7.4, 100 mM NaCl, 0.3% NP-40, 2 mM PMSF, protease inhibitor cocktail, 1 tablet/mL) and eluted the immune complexes into SDS-loading buffer. Western transfer and immunoblotting were performed as described previously and using anti-Flag (Sigma, Catalog #: F1804, Clone: M2), anti-TGF-β1 (Abcam, Catalog #: ab179695, Clone: EPR18163), anti-TGF-β1/β2/β3 (R&D Systems, Catalog #: MAB1835-100, Clone: 1D11), and anti-His_6_ (Abcam, Catalog #: ab18184, Clone: HIS:H8) antibodies. *Co-IP of TGF-β2 and -β3 by fortilin*: For the Co-IP of TGF-β2 and -β3 by fortilin, the same procedure was repeated using rh-TGF-β2 (R&D Systems, Catalog #: 302-B2-010/CF), rh-TGF-β3 (R&D Systems, Catalog #: 243-B3-010/CF), and Buffer B for the wash, which was performed five times instead of four times.

### Generation of TGF-β1-rich sera

De-identified human platelet units were obtained from the University of Washington Blood Bank (Seattle, WA, USA) and stored at –80 °C until used in the experiment. After the unit was thawed, we added thrombin at the final concentration of 0.5 unit/mL, immediately mixed the solution, incubated it at room temperature for 10 min with gentle shaking, centrifuged the mixture at 10,000 × *g*. We collected the separated supernatant, labeled it “sera”, and stored it at −80 °C.

### Immunodepletion of fortilin or TGF-β1 from human sera

Mouse anti-TGF-β monoclonal antibody (α-TGF-β mAb, Catalog #: MAB1835-100, Clone 1D11, R&D Systems) and α-fortilin mAb were coupled to Dynabeads® M-280 tosylactivated magnetic beads (Thermo Fisher Scientific) according to the manufacturer’s instructions. We incubated 180 µL of the platelet-rich sera with 100 µL each of magnetic beads (20 mg/mL) coupled with either α-TGF-β or α-fortilin mAbs for 2 h at 37 °C, immobilized the beads with a magnet, and transferred the supernatants (the sera immunodepleted of fortilin and TGF-β) to fresh microfuge tubes. We repeated the process twice before we stored the samples at –80 °C until the ELISA experiments.

### ELISA

*TGF-β1 ELISA*: For ELISA of TGF-β1, the TGF-β1 human ELISA kit was purchased from Abcam (Catalog #: ab100647) and used according to the manufacturer’s instructions. We first applied 100 µL of control and study sera to the wells of a 96-well strip plate that had already been pre-coated with α-TGF-β1 capturing antibody. After incubating the plate for 2.5 h at room temperature with gentle shaking and washing it extensively with wash buffer provided by the manufacturer, we added 100 µL of biotinylated α-TGF-β1 detection antibody and incubated it for 1 h at room temperature. After another extensive wash, we added horse-radish-peroxidase (HRP)-conjugated streptavidin solution to each well and incubated it for 45 min at room temperature with gentle shaking. After a final extensive wash, we added 3,3′,5,5′-tetramethylbenzidine (TMB) substrate solution to each well, incubated the plate in the dark for 30 min at room temperature with gentle shaking, added 50 µL of stop solution to each well, and obtained the absorbance at 450 nm (Abs_450_), using the Multilabel Plate Reader (Victor 3 V, Model 1420, Perkin Elmer, Waltham, MA, USA). *Fortilin ELISA*: For ELISA of fortilin, we first biotinylated α-fortilin mAb using the Lighting-Link® Biotinylation Kit (Catalog #: ab201795, Abcam). We then coated the wells of a 96-well plate (Clear Flat Bottom Polystyrene High Bind Microplate, Corning; Catalog #: 9018) by adding 200 µL of 1 µg/mL α-fortilin mAb to the wells. We incubated the plate for 1 h at 37 °C and then washed it three times with wash buffer (PBS with 150 mM NaCl, 0.05% Tween 20). We then applied 100 µL of control and study sera to the wells of the plate. After incubating the plate for 2.5 h at room temperature with gentle shaking and washing it extensively, we added 100 µL of biotinylated α-fortilin detection antibody and incubated it for 1 h at room temperature. After extensive washing, we added HRP-streptavidin solution (Abcam, Catalog #: ab7403) to each well and incubated the plate for 45 min at room temperature with gentle shaking. Finally, after extensive washing, we added TMB substrate solution (Abcam, Catalog #: ab171522) to each well, incubated the plate in the dark for 3 min at room temperature with gentle shaking, added 100 µL of stop solution (Abcam, Catalog #: ab171529) to each well, and obtained the absorbance at 450 nm. *Modified ELISA to detect the fortilin-TGF-β1 interaction* in vivo: To evaluate the in vivo complexing between TGF-β1 and fortilin, we first applied 100 µL of these samples to the wells of a 96-well TGF-β1 human ELISA strip plate (Abcam, Catalog #; 100647) that had already been pre-coated with α-TGF-β1 capturing antibody. After incubating the plate for 2.5 h at room temperature with gentle shaking and washing it extensively, we added 100 µL of biotinylated α-fortilin detection antibody and incubated it for 1 h at room temperature. After extensive washing, we added HRP-streptavidin solution to each well and incubated the plate for 45 min at room temperature with gentle shaking. Finally, after extensive washing, we added TMB substrate solution (Abcam) to each well, incubated it in the dark for 30 min at room temperature with gentle shaking, added 50 µL of stop solution (Abcam) to each well, and obtained the absorbance at 450 nm.

### MILLIPLEX® multiplex immunoassays to detect mouse serum TGF-β1, TGF-β2, and TGF-β3

We determined the serum concentrations of TGF-β1, TGF-β2, and TGF-β3 by diluting mouse serum samples by adding 1 part of serum to 29 parts of Sample Diluent (Catalog #: LTGF-SD, Millipore Sigma, Burlington, MA, USA) and subjecting them to MILLIPLEX® multiplex immunoassays (Catalog #: TGFBMAG-64K-03, Millipore Sigma), according to the manufacturer’s instructions.

### Co-purification of fortilin by column-based affinity purification of TGF-β1

We performed all steps in 4 °C. To test if affinity-purified TGF-β1 is bound to fortilin, we first packed a gravity chromatography column (Bio-Rad Poly-Prep Chromatography Columns) with 1 mL agarose beads conjugated to anti-TGF-β1 antibody (Santa Cruz Biotechnology) and equilibrated it with 10-column volume (CV) of Wash Buffer (phosphate-buffered saline (PBS)) (Figs. S1b-1). Next, we loaded the column with 10 mL of platelet-rich sera diluted 10-fold in Wash Buffer (Figs. S1b-2) and collected the entire flow through (FT). Next, we washed the column with 10 CV of Wash Buffers 5 times (Figs. S1b-3). We collected flow throughs (WASH-1, -2, -3, -4, and -5) and monitored their protein concentrations using NanoDrop™ One (ThermoFisher Scientific). Preliminary experiments had showed that protein concentrations of WASH-4 and WASH-5 were barely detectable using NanoDrop One (Thermo Scientific). We then put the bottom cap onto the column, added 1 mL of Elution Buffer (0.1 M Glycine-HCl, pH = 3.0), incubated for 1 min, and collected the eluate (EL-1). We repeated the step two more times to collect EL-2 and EL-3 (Figs. S1b-4). We pooled together EL-1, EL-2, and EL-3 and concentrated it to 73 µL (ELUATE), using a protein concentrator spin column (Amicon Ultra-0.5 centrifugal filter unit, Millipore Sigma, Burlington, MA, USA; Catalog #: UFC5010). Finally, we subjected 37.5 µL each of platelet-rich sera (INPUT), FT, WASH-1, WASH-2, WASH-4, WASH-5, and ELUATE to western blot analysis using anti-fortilin (Abcam, EPR5540) and anti-TGF-β1 (Abcam EPR18163).

### BLI analysis

The interaction between fortilin and TGF-β1 was evaluated using the BLItz System (ForteBio) as described previously^5^. First, rh-fortilin protein was biotinylated by mixing it with 2 mM NHS-PEG4-biotin solution and incubating the mixture for 30 min at room temperature. The biotinylated protein was then purified to remove free biotin using Zeba™ Spin Desalting Columns (Thermo Fisher Scientific). Next, the biotinylated fortilin was immobilized on streptavidin-coated biosensors (ForteBio) at a concentration of 30 μg/mL in PBS for 600 s. After fortilin-coated biosensors were buffer-exchanged in PBS for 30 s, various concentrations of rh-TGF-β1 (OriGene; Catalog #: TP300973) were added for 180 s to evaluate the association between the two proteins. Finally, rh-TGF-β1 protein solution was replaced by PBS for 180 s to evaluate their dissociation. The binding data were processed, and a dissociation constant (K_d_) was calculated using BLItz analysis software based on two independent experiments. The same procedure was performed for the LAP-TGF-β1 protein (R&D Systems), except the K_d_ was calculated based on three independent experiments.

### SPR analysis

All experiments were performed using a Biacore 3000 (Uppsala, Sweden) and carried out at 25 °C using HBS-EPS (0.01 M HEPES, 0.5 M NaCl, 3 mM EDTA, 0.005% (v/v) Tween 20, pH 7.4) as running buffer. The experimental flow rate was 20 μL/min. Different forms of rh-fortilin were immobilized onto three flow channels (FCs) of a CM5 chip using amine-coupling chemistry as follows: FC2: 7060 RU N-His_6_-fortilin; FC3: 3223 RU C-His_6_-fortilin; FC4: 3667 RU strep-tag-fortilin. To scout for binding conditions, rh-TGF-β1 produced in-house was injected at a concentration of 100 nM over each channel. To obtain binding rates, rh-TGF-β1 was injected over N-His_6_-fortilin at the indicated concentrations. Binding rates were calculated by fitting data to a 1:1 Langmuir interaction model with mass transport limitation using BiaEvaluation software (Biacore). K_d_s were determined by calculating the ratio of binding and dissociation rate constants. For inhibition analysis, 100 nM rh-TGF-β1 was pre-incubated with indicated concentrations of the inhibitor rh-TGFβRII-Fc and injected over experimental and control flow channels^19^. After each binding cycle, the fortilin-coupled surface was regenerated to base line by injecting 20 µL of 1 M NaCl.

### Luciferase assay using HEK293^SBE-Luc^ cells

We transiently transfected HEK293 cells with the SBE-luciferase vector (pGL4.48 [luc2P/SBE/Hygro], Promega, Madison, WI, USA) along with the Renilla luciferase control reporter vector (pRL, Promega), which allowed us to normalize firefly luciferase activities according to transfection efficiency. The following day, we treated the transfected HEK293 cells with rh-TGF-β1 (1 nM) or vehicle (PBS) in the presence and absence of step-tag-fortilin (3 nM) in quadruplicate, incubated the samples for 18 h at 37 °C, and subjected the cells to the Dual-Glo-Luciferase Assay System (Promega) according to the manufacturer’s instructions. We calculated relative luciferase activity (FLU/RLU) by dividing firefly luciferase units (FLU) by Renilla luciferase units (RLU) and expressed the results as A.U. We then normalized FLU/RLU of the cells treated with rh-TGF-β1 to that of the cells treated with vehicle in either the presence or absence of fortilin.

### SEAP reporter assay using MFB-F11^SBE-SEAP^ cells

MFB-F11 cells^21,22^ were a kind gift from Dr. Tony Wyss-Coray (Stanford University, Stanford, CA, USA). The cells were generated at the Wyss-Coray lab by stably transfecting mouse embryonic fibroblasts from *Tgfb1*^*−/−*^ mice with a synthetic promoter element containing 12 CAGA boxes fused to a SEAP reporter gene^22^. The cells were kept and propagated in DMEM supplemented with 10% FBS. For SEAP reporter assays, we first acclimated murine MFB-F11 cells in FibroLife Fibroblast Serum Free Medium (LifeLine Cell Technology, Oceanside, CA, USA) with 1% FBS. We seeded MFB-F11 cells in a 96-well plate at 50,000 cells per well (*N* = 6 each) and incubated them for 24 h. The next morning, we replaced the medium with 100 µL of FibroLife without FBS and incubated the cells at 37 °C for 2 h. We then stimulated the cells with recombinant mouse (rm) TGF-β1 (R&D Systems, Catalog #: 7666-MB/CF; 156 pM or 2000 pg/mL) pre-incubated with strep-tagged rh-fortilin (either 19.5 or 195 nM), strep-tagged recombinant luciferase (either 19.5 or 195 nM), or anti-TGF-β1 monoclonal antibody (R&D Systems, Catalog #: MAB1835-100, Clone: 1D11) for 24 h at 37 °C. We then subjected 40 µL of supernatants to the Phospha-Light™ SEAP Reporter Gene assay (Thermo Fisher Scientific, Catalog #: T1015) according to the manufacturer’s instructions.

### Smad3 phosphorylation assay by western blot analysis and ELISA

We seeded 0.7 × 10^6^ FibroLife-acclimated MFB-F11^SBE-SEAP^ cells in each well of 6-well plates and incubated them for 24 h. We then replaced the medium with 100 µL of FibroLife without FBS and incubated the cells at 37 °C for 2 h. Next, we stimulated the cells for 45 min at 37 °C with rm-TGF-β1 (2 ng/mL or 156 pM) that had been pre-incubated with either fortilin or control luciferase proteins. We harvested the cells for either standard western blot analysis or the *P*-Smad3 (pS423/S425) ELISA, with the latter following the manufacturer’s instructions (Abcam, Catalog #: ab186038).

### Quantitative real-time PCR

Mouse embryonic fibroblast C3H10T1/2 cells were seeded in a 12-well plate at a density of 1 × 10^5^ cells/well and cultured overnight. Cells were starved with DMEM containing 0.1% FBS for 48 h, followed by incubation with fortilin (0.5 µg/mL), TGF-β1 (1 ng/mL), or their mixture for 8 h. All proteins, individually or in mixture, were incubated in the starving medium at room temperature for 30 min before treatment. Total RNA was isolated at 8 h after treatment using TRIzol (Invitrogen, Carlsbad, CA, USA). cDNA was synthesized using the iScript™ cDNA Synthesis Kit (Bio-Rad). PCR was performed using the All-in-One™ qPCR mix (GeneCopoeia, Rockville, MD, USA) on an AriaMx Real-time PCR system (Agilent, Santa Clara, CA, USA) following the manufacturer’s instructions. Cyclophilinm (CYP) was used as an internal reference. The primers (Integrated DNA Technologies, Coralville, IA, USA) were as follows: *CNN1*, Forward: 5′-GGATGTGACAGCAGCGTTTG-3′, Reverse: 5′-GGCCCCAAGACTCCAATGAT-3′; *α-SMA*, Forward: 5′-AATGGCTCTGGGCTCTGTAAG-3′, Reverse: 5′-CACGATGGATGGGAAAACAGC-3′; *SM22α*, Forward: 5′-GGTCCATCCTACGGCATGAG-3′, Reverse: 5′-CCTACATCAGGGCCACACTG-3′; *CYP*, Forward: 5′-CAGACGCCACTGTCGCTTT-3′, Reverse: 5′-TGTCTTTGGAACTTTGTCTG-3′. Gene expression was calculated using the 2^–∆∆Ct^ method. The experiments were independently repeated three times.

### Analysis and alignments of TGF-β isoform sequences

TGF-β1, -β2, and -β3 sequences were obtained from Mittl et al.^72^. Unipro UGENE v34.0 (Unipro, Novosibirsk, Russia) was used to align their sequences and visualize the alignment according to the developers’ instructions^73^.

### Computational molecular docking of fortilin, TGF-β1, and TGFβRII

Three-dimensional protein structural models of fortilin (PDB ID: 2HR9), TGF-β1 (PDB ID: 3KFD), and the TGF-β1:TGFβRII complex (PDB ID: 5TY4) for docking experiments were obtained from the Research Collaboratory for Structural Bioinformatics (RCSB) Protein Data Bank (PDB)^29,74–76^. Protein–protein docking of fortilin with TGF-β1 and TGF-β1:TGFβRII was performed using the ClusPro server utilizing the PIPER docking algorithm^33,34,77,78^. For the docking procedures, the center-of-mass (COM) of the receptor TGF-β1 protein (or TGF-β1:TGFβRII) was fixed, and the fortilin position was sampled at 70,000 possible rotational orientations about the COM. At each rotational position, the ligand translational position was sampled at a resolution of 1 Å to find the corresponding lowest-energy conformation. In total, the algorithm sampled ~10^9^ possible relative orientations of the proteins. Interaction energies (or scores) for each conformation were calculated using an electrostatic-favored weighting due to the overall high density of charged residues on the protein surfaces. The docking procedure is followed by clustering of similarly oriented structures of the 1000 lowest-energy structures. The interfacial root mean squared deviation (IRMSD) of backbone atoms are calculated for each structure, and that with the largest number of neighboring structures within a 9 Å radius is defined as the center of the first cluster. All protein–protein orientations within the 9 Å IRMSD are considered part of the first cluster and removed from the population. The procedure is repeated for the remaining population until all docked structures are clustered. The populations of the resulting clusters are proportional to the thermodynamic probabilities of finding the proteins in each specific binding orientation. The scores are calculated from molecular mechanics (CHARMM force field) for the structures obtained from docking.

### Statistics and reproducibility

The degree of the spread of data was expressed by the standard deviation (mean ± SD). Student’s *t*-test was used to compare the means of two groups. To compare the means of three groups, we used one-way ANOVA with Fisher’s pairwise comparison. *P* < 0.05 was considered to be statistically significant. *P* < 0.10 was considered to show a trend toward statistical significance. The numbers of biological replicates used in in vivo experiments were determined by (i) power analysis, assuming an α error rate of 0.05, β error rate of 0.20, and expected difference of 25% and using Minitab 17 (State College, PA, USA) or (ii) our previous dataset and experience from similar experiments performed in the past.

### Reporting summary

Further information on research design is available in the Nature Research Reporting Summary linked to this article.

## Supplementary information

Supplementary Information

Description of Additional Supplementary Files

Supplementary Data 1

Reporting Summary

## Data Availability

The authors declare that the data supporting the findings of this study are available within the paper and its supplementary information files (Figs. S1–S10 and Supplementary Data 1). All relevant data are available from the authors upon request.
