# Supplementary material for: Fortilin interacts with TGF-β1 and prevents TGF-β receptor activation
Source: Commun Biol. 2022 Feb 23;5:157. doi: 10.1038/s42003-022-03112-6 (PMC8866402; doi:10.1038/s42003-022-03112-6)
Supplement: Supplementary file 1 — Supplementary Information [file 42003_2022_3112_MOESM1_ESM.pdf]

Fortilin interacts with TGF-β1 and prevents TGF-β receptor activation

SUPPLEMENTARY INFORMATION

Decha Pinkaew[1], Erik Martinez-Hackert[2], Wei Jia[3], Matthew King[4], Fei Miao[1,6], Nicole R. Enger[1], Runglawan Silakit[1], Kota Ramana[5], Shi-You Chen[3], and Ken Fujise[1,*]

[1]Division of Cardiology, Department of Medicine, University of Washington, Seattle, WA 98109, USA; [2]Department of Biochemistry and Molecular Biology, Michigan State University, East Lansing, MI 48824, USA; [3]Department of Surgery, University of Missouri, Columbia, MO 65212, USA; [4]Department of Chemistry and Biochemistry, Boise State University, Boise, ID 83725, USA; [5]Department of Biochemistry, Noorda College of Osteopathic Medicine, Provo, UT 84606, USA; [6]Department of Pathology and Laboratory Medicine, University of Pennsylvania, Philadelphia, PA 19104, USA (present address).

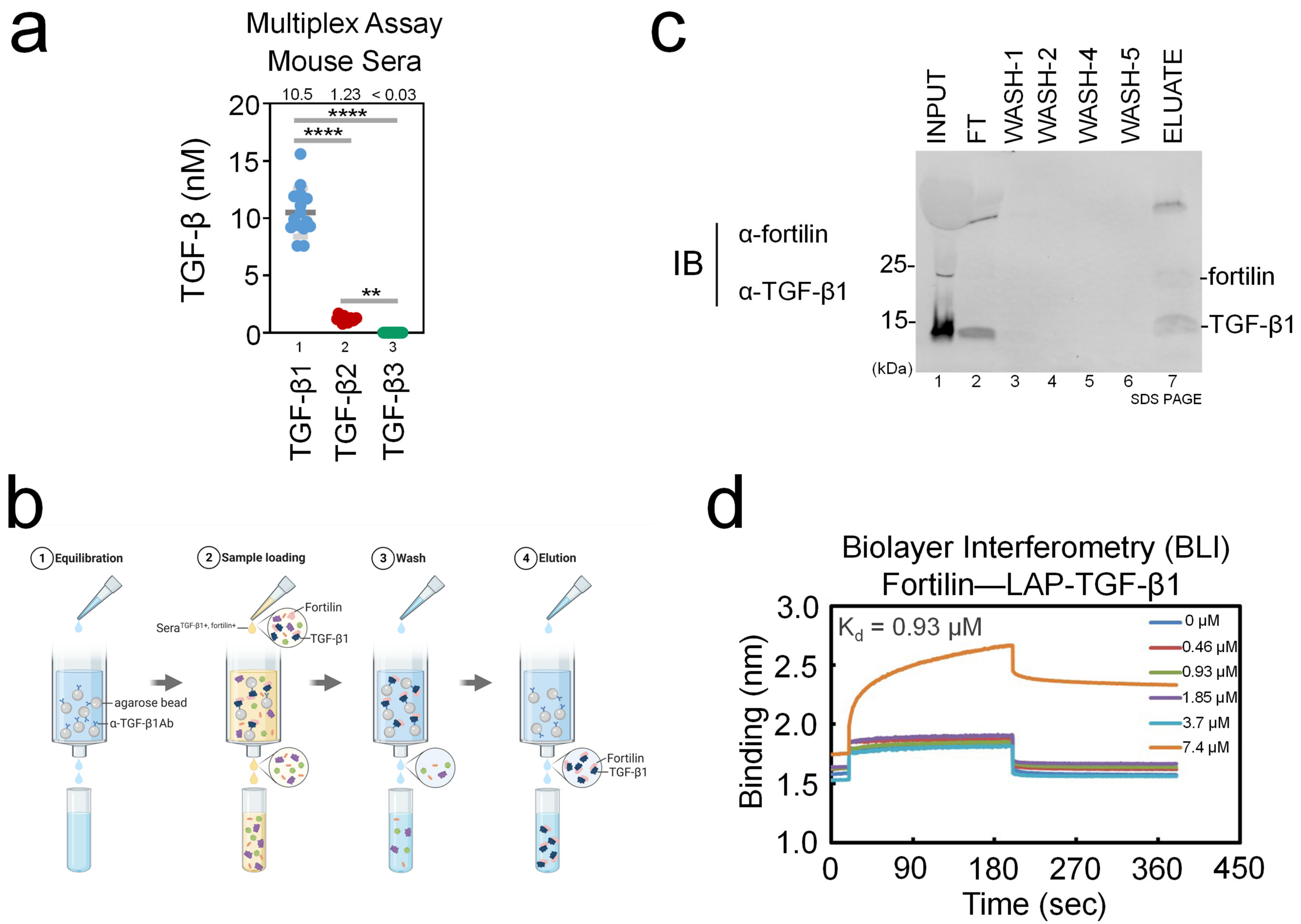

Figure S1. **Serum concentrations of TGF-βs, column co-purification of fortilin and TGF-β1, and biolayer interferometry (BLI) of fortilin and the latency-associated peptide (LAP).** Abbreviations: IB, immunoblot, α-fortilin, anti-fortilin antibody; α-TGF-β1, anti-TGF-β1 antibody; INPUT, sera generated from platelet-rich plasma ($Sera^{TGF\text{-}\beta1+,\ fortilin+}$); FT, flow-through; WASH, flow-through from wash; ELUATE, collected and concentrated eluate; BLI, Biolayer intereferometry; K_d, dissociation constant. N, the number of biological replicates; **, P < 0.01, ****, P < 0.001 by one-way ANOVA with Fisher's pairwise comparison. **(a) Multiplex Assay of TGF-βs in mouse sera.** Serum concentrations of TGF-β1, -β2, and -β3 were determined by a multiplex assay system. TGF-β3 levels were all below the detection limit (0.023 nM) of the assay system. N = 15 each. **(b) Experimental scheme of column co-purification of fortilin and TGF-β1.** After packing and equilibrating a gravity chromatography column with agarose beads conjugated to α-TGF-β1 antibody (**1**), we loaded it with $Sera^{TGF\text{-}\beta1+,\ fortilin+}$ (**2**), extensively washed it (**3**), and eluted the bound proteins (**4**). **(c) Western blot analyses of the input, flow-through, wash flow-throughs and eluate.** The eluant contained both fortilin and TGF-β1 (ELUATE), suggesting that affinity-purified TGF-β1 was bound to fortilin and that fortilin and TGF-β1 form a complex in vivo in normal human sera. **(d) BLI.** BLI showed the specific binding of fortilin to the LAP-TGF-β1 protein at K_d of 0.93 μM, substantially weaker interaction than one between fortilin and mature TGF-β1 (94.5 nM).

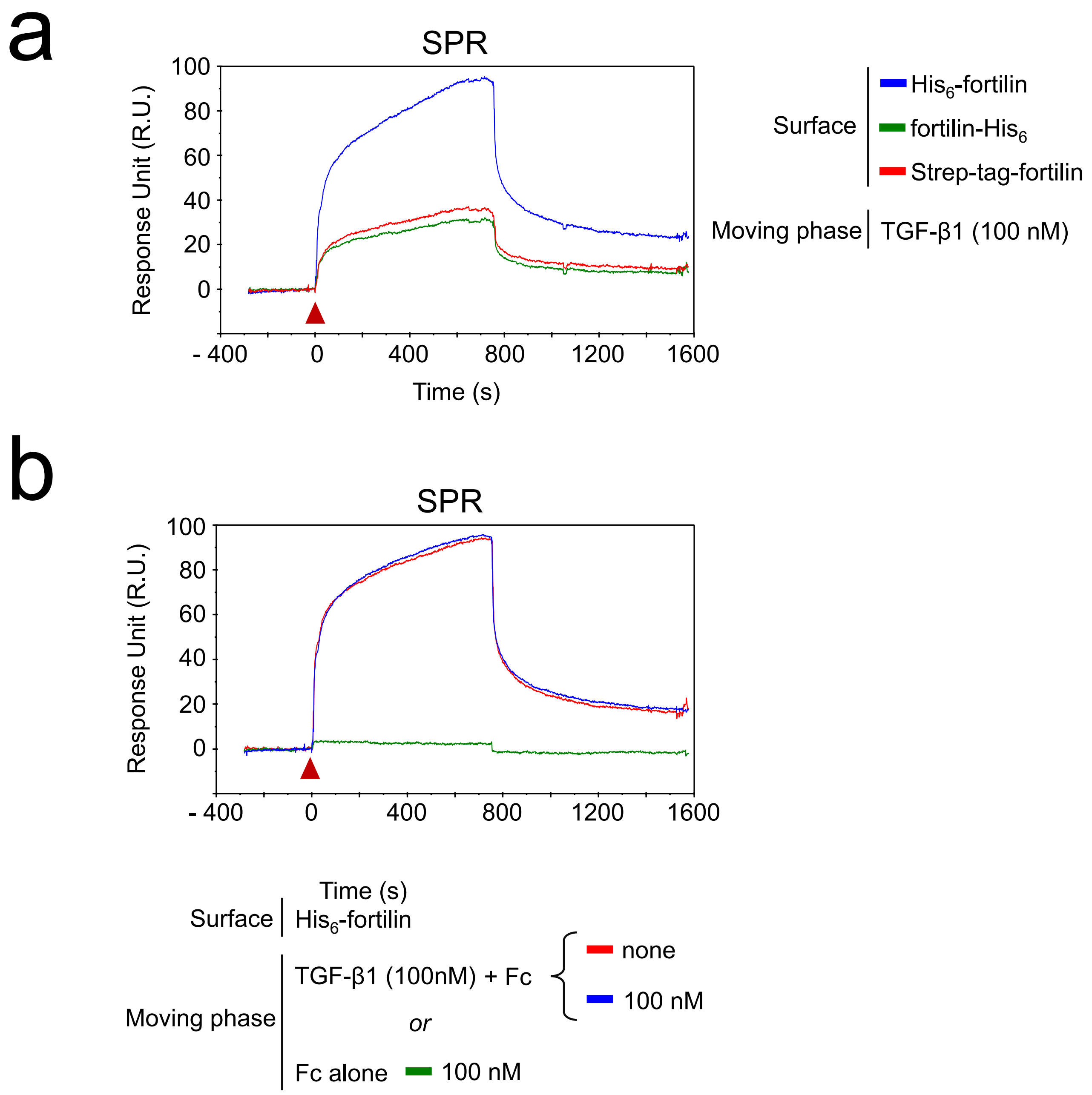

Figure S2. **SPR shows the specific interaction between fortilin and TGF-β1.** Abbreviations: SPR, surface plasmon resonance; His$_6$-fortilin, recombinant fortilin with N-terminus hexa-his-tag; fortilin-His$_6$, recombinant fortilin with C-terminus hexa-his-tag; strep-tag-fortilin, recombinant fortilin with N-terminus strep-tag; Fc, the Fc portion of the IgG. **(a) Interaction between various recombinant fortilins and TGF-β1 as assessed by SPR.** His$_6$-fortilin, fortilin-His$_6$, or strep-tag-fortilin were conjugated to the SPR chip surface, and TGF-β1 was injected onto the chip surface at 100 nM. The data suggest that TGF-β1 binds all three forms of recombinant fortilins. **(b) Lack of interaction between Fc and fortilin/TGF-β1.** His$_6$-fortilin was conjugated to the SPR chip surface and (i) TGF-β1 alone, (ii) TGF-β1 and Fc, or (iii) Fc alone was injected onto the surface. The data suggest that fortilin binds TGF-β1, but not Fc, and that Fc alone does not block the binding of fortilin to TGF-β1.

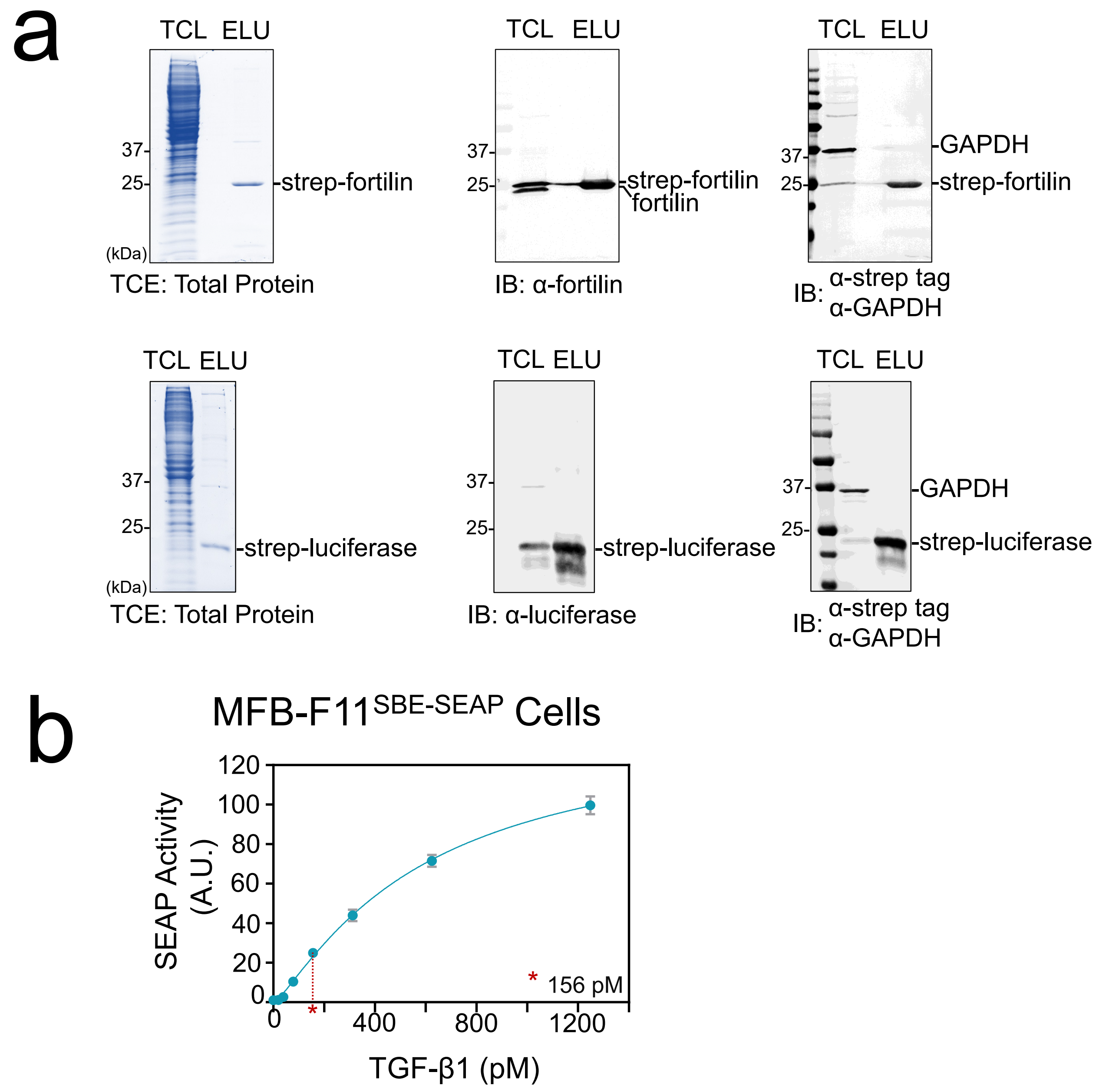

Figure S3. **Expression, purification, and characterization of strep-tagged fortilin and luciferase.** Abbreviations: TCL, total cell lysate; ELU, eluent; TCE, 2,2,2-trichloroethylene; IB, immunoblot; α-fortilin, anti-fortilin antibody; α-GAPDH, anti-glyceraldehyde 3-phosphate dehydrogenase antibody; α-strep-tag; anti-strep-tag antibody; α-luciferase, anti-luciferase antibody. A.U., arbitrary unit; SBE-SEAP, a vector containing the Smad2/3 binding element fused to the secreted embryonic alkaline phosphatase cDNA; MFB-F11$^{SBE\text{-}SEAP}$ cells, immortalized mouse embryonic fibroblasts from *Tgfb1*$^{-/-}$ mice that stably harbor the SBE-SEAP construct; *, concentration of TGF-β1 used for the main experiments. **(a) Mammalian cell expression and column purification of strep-tag fortilin and luciferase proteins.** 293T cells were transiently transfected with the mammalian expression plasmid containing either strep-fortilin or strep-luciferase cDNA. Total cell lysates were subjected to purification using the Strep-Tactin® XT Superflow® high-capacity column. Aliquots from total cell lysates and eluents were analyzed by western blot analysis. **(b) A wide dynamic range of SEAP response to TGF-β1 in MFB-F11$^{SBE\text{-}SEAP}$ cells.** We stimulated MFB-F11$^{SBE\text{-}SEAP}$ cells with various concentrations of TGF-β1, sampled conditioned media, and determined SEAP activities. Based on the data, we decided to use 156 pM (*) of TGF-β1 for our main experiments.

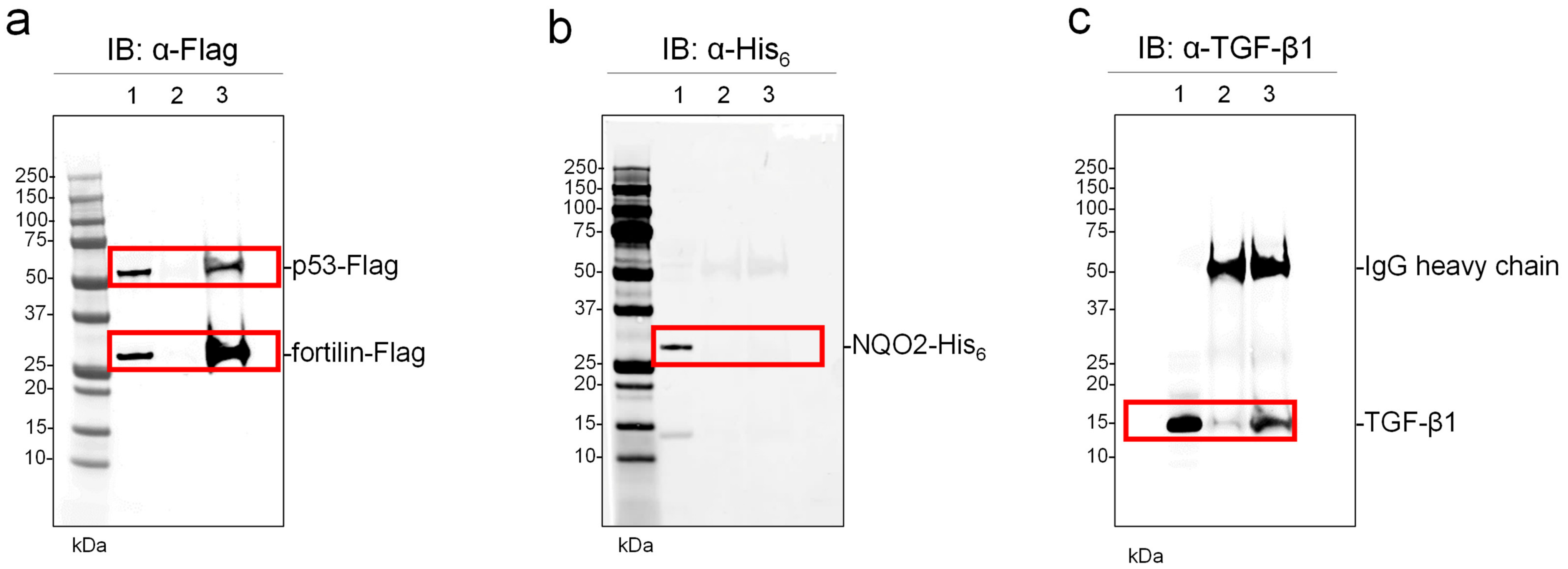

Figure S4. **The Full unprocessed images of blots used for Fig. 1a.** Abbreviation: IB, immunoblot. Red frames show the protein bands used in the figure. **(a)** The blot was used for fortilin-Flag and p53-Flag of Fig.1a. **(b)** The blot was used for NQO2-His6 of Fig.1a. **(c)** The blot was used for TGF-β1 of Fig. 1a. **(b, c)** The same membrane with mixed mouse α-His_6 and rabbit α-TGF-β1 antibodies. The 2nd antibodies are anti-rabbit IRDye-800 (Green) and anti-mouse IRDye-680LT (Red) were detected using the Bio-Rad ChemiDoc MP Imaging Systems with Green and Red channels, respectively. Molecular weight markers is Precision Plus Protein™ All Blue Prestained Protein Standards (Bio-Rad#1610373), which was easily detected in Red channel **(b)** but barely seen in Green channel even with high very exposure **(c)**. Hence, the markers in α-TGF-β1 membrane was marked using corresponding markers from α-His_6 membrane with the fade bands of IgG heavy chain as a guide.

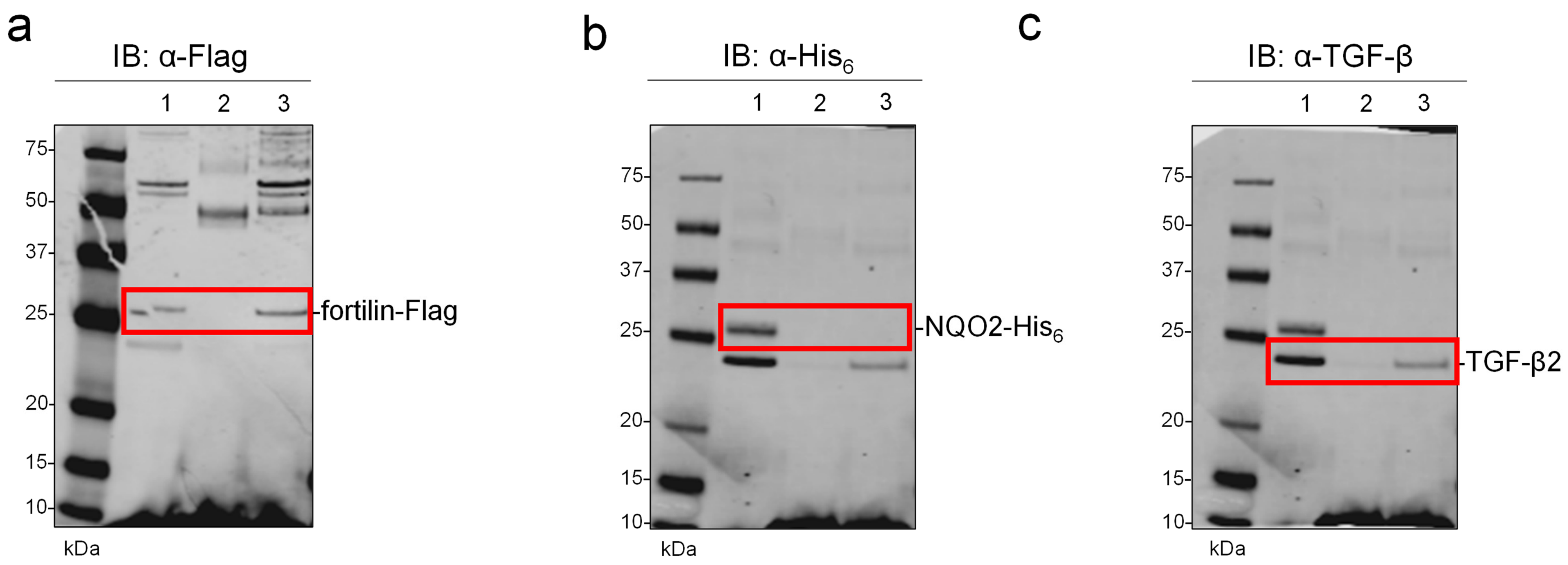

Figure S5. **The Full unprocessed images of blots used for Fig. 1b, the left panel.** Abbreviation: IB, immunoblot. Red frames show the protein bands used in the figure. **(a)** The blot was used for fortilin-Flag of the TGF-β2 panel of Fig.1b. **(b)** The blot was used for NQO2-His6 of the TGF-β2 panel of Fig.1b. **(c)** The blot was used for TGF-β2 of the TGF-β2 panel of Fig.1b.

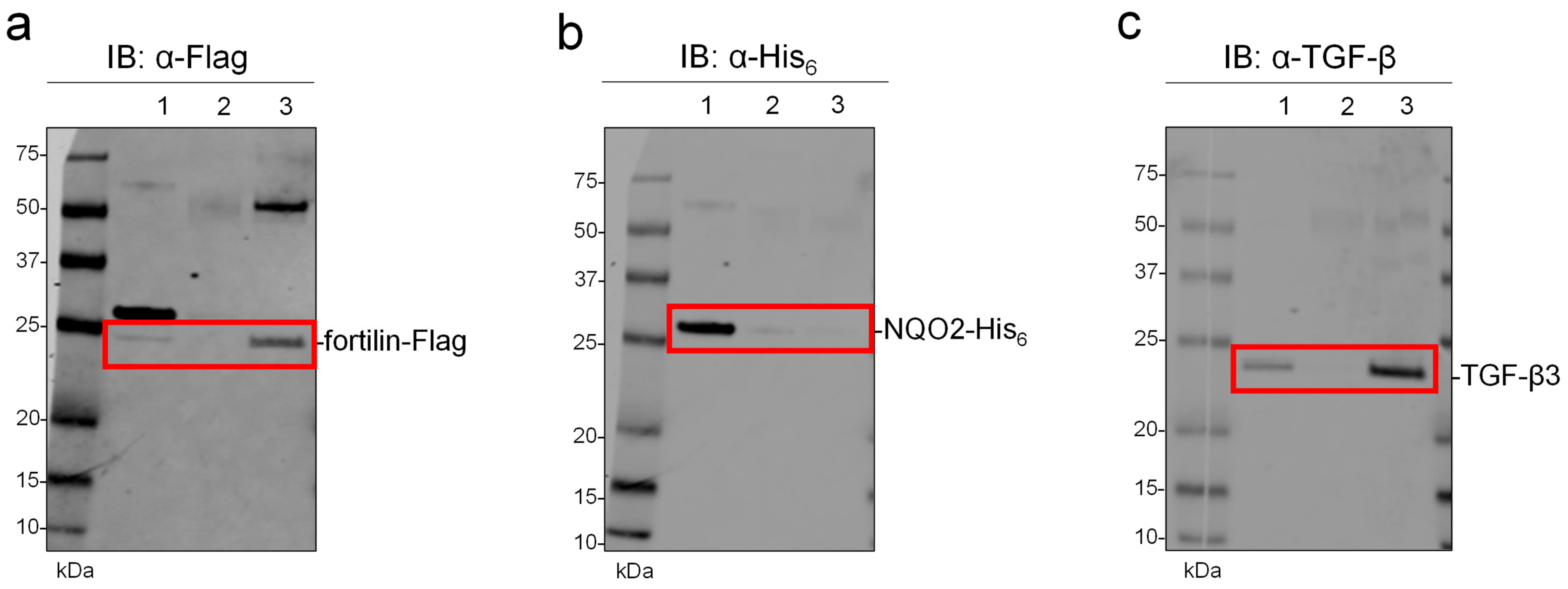

Figure S6. **The Full unprocessed images of blots used for Fig. 1b, the right panel.** Abbreviation: IB, immunoblot. Red frames show the protein bands used in the figure. **(a)** The blot was used for fortilin-Flag of the TGF-β3 panel of Fig.1b. **(b)** The blot was used for NQO2-His6 of the TGF-β3 panel of Fig.1b. **(c)** The blot was used for TGF-β3 of the TGF-β3 panel of Fig.1b.

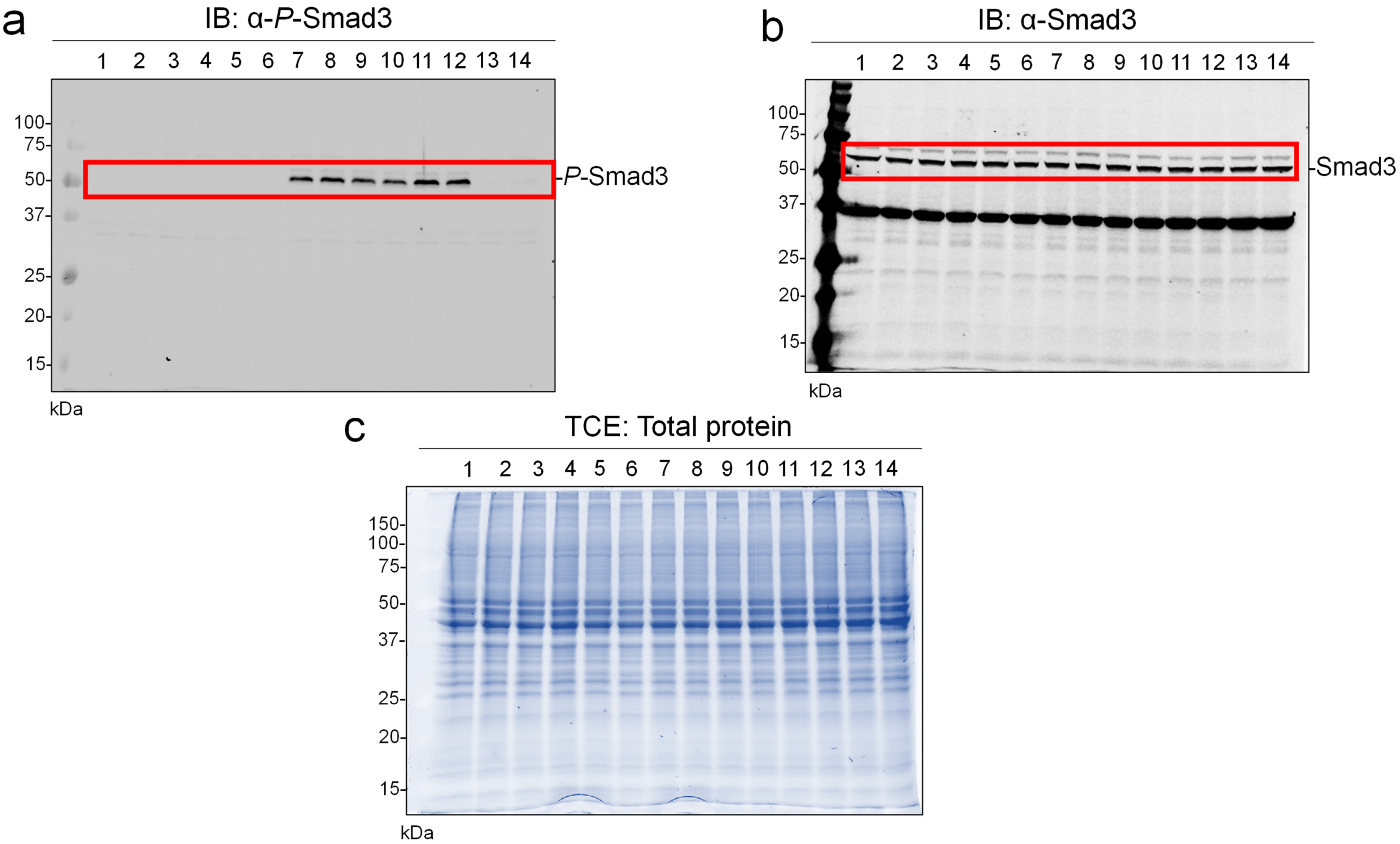

Figure S7. **The Full unprocessed images of blots and a gel used for Fig. 4a.** Abbreviation: IB, immunoblot; TCE, 2,2,2-Trichloroethanol which binds to the aromatic amino acids of protein samples and the proteins fluoresce under UV light. **(a)** The blot used for the P-Smad3 image of Fig. 4a. **(b)** The blot used for the Smad3 image of Fig. 4a. **(c)** The gel used for total proteins in Fig. 4a. Precision Plus Protein™ All Blue Prestained Protein Standards (Bio-Rad#1610373) appear as white bands in TCE-stained gel.

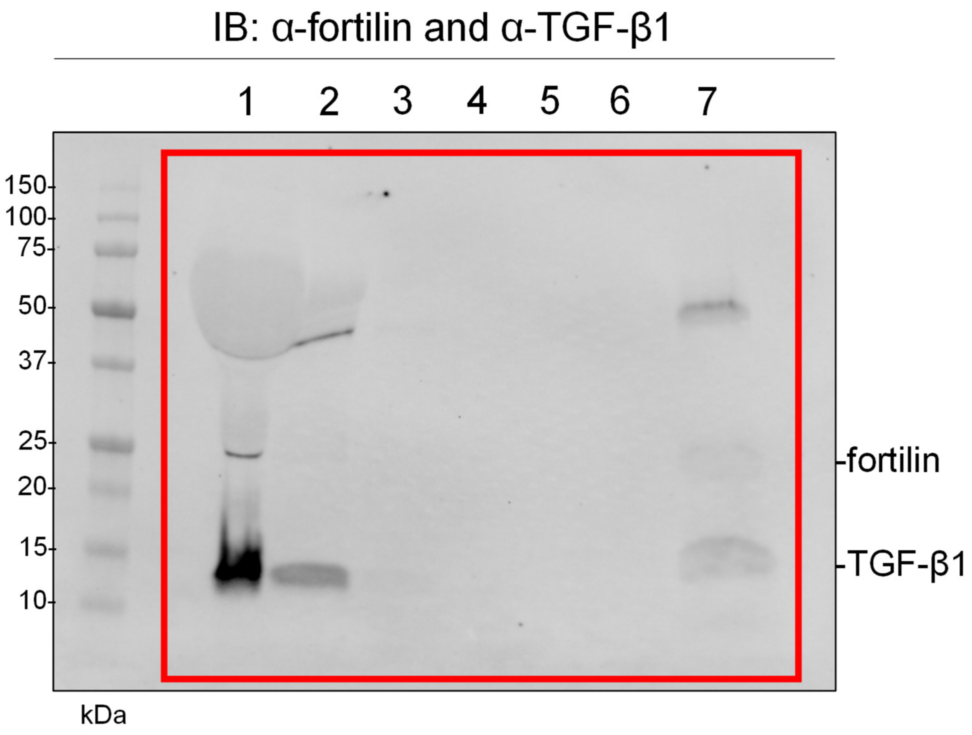

Figure S8. **The Full unprocessed image of a blot used for Fig. S1c.** Abbreviation: IB, immunoblot.

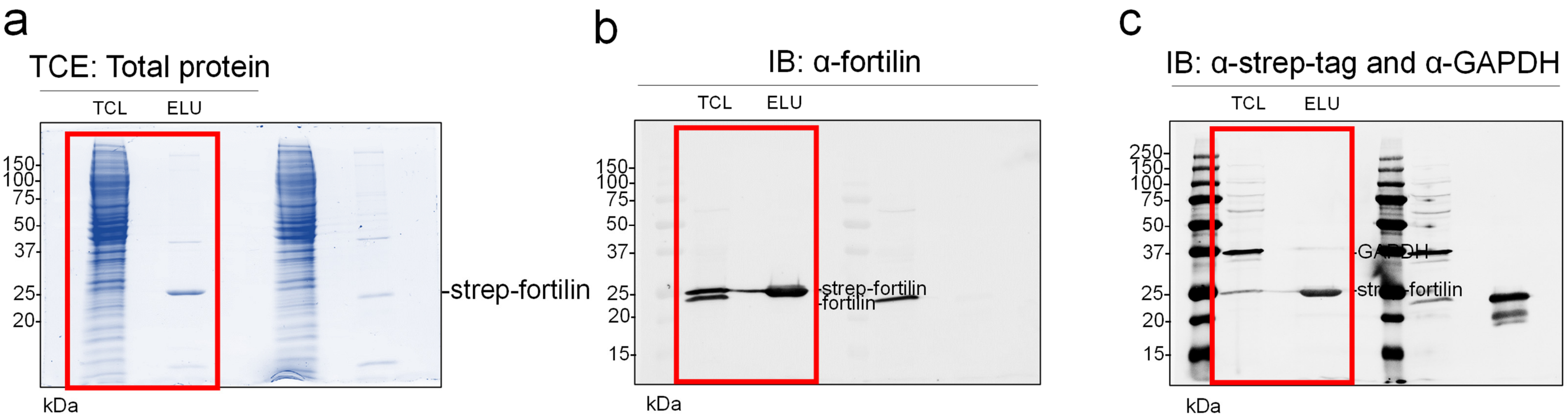

Figure S9. **The Full unprocessed images of blots and a gel used for Fig. S3a, the upper panel.** Abbreviation: IB, immunoblot; TCE, 2,2,2-Trichloroethanol which binds to the aromatic amino acids of protein samples and the proteins fluoresce under UV light. **(a)** The gel used for total proteins in the upper left panel of Fig. S3a. Precision Plus Protein™ All Blue Prestained Protein Standards (Bio-Rad#1610373) yield white bands in TCE-stained gels. **(b)** The blot used for the immunoblot image in the upper middle panel of Fig. S3a. **(c)** The blot used for the immunoblot image in the upper right panel of Fig. S3a. **(b, c)** The same membrane was probed with the mixture of mouse α-strep-tag, mouse α-GAPDH and rabbit α-fortilin antibodies. The 2nd antibodies were anti-rabbit IRDye-800 (Green) and anti-mouse IRDye-680LT (Red), which were detected using the Bio-Rad ChemiDoc MP Imaging Systems with Green and Red channels, respectively. Precision Plus Protein™ All Blue Prestained Protein Standards (Bio-Rad#1610373) were used as molecular weight markers which were detected in Red channel but did not yield strong signal in Green channel even with high very exposure.

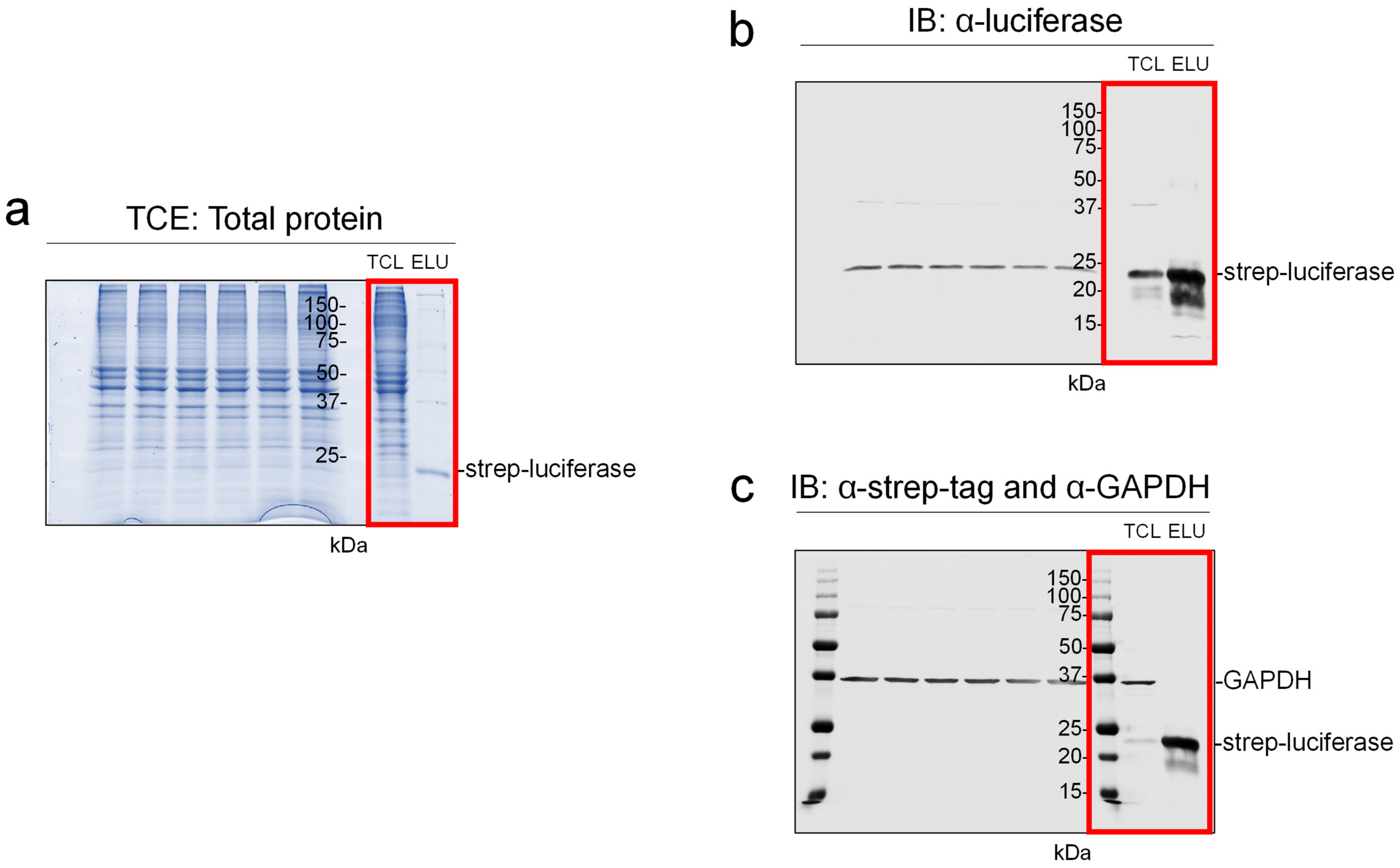

Figure S10. **The Full unprocessed images of blots and a gel used for Fig. S3a, the lower panel.** Abbreviation: IB, immunoblot; TCE, 2,2,2-Trichloroethanol which binds to the aromatic amino acids of protein samples and the proteins fluoresce under UV light. **(a)** The gel used for total proteins in the lower left panel of Fig. S3a. Precision Plus Protein™ All Blue Prestained Protein Standards (Bio-Rad#1610373) yield white bands in TCE-stained gels. **(b)** The blot used for the immunoblot image in the lower middle panel of Fig. S3a. **(c)** The blot used for the immunoblot image in the lower right panel of Fig. S3a. **(b, c)** The same membrane was probed with the mixture containing mouse α-strep-tag, mouse α-GAPDH and rabbit α-luciferase antibodies. The 2nd antibodies were anti-rabbit IRDye-800 (Green) and anti-mouse IRDye-680LT (Red), which were detected by the the Bio-Rad ChemiDoc MP Imaging Systems with Green and Red channels, respectively. Precision Plus Protein™ All Blue Prestained Protein Standards (Bio-Rad#1610373) were used as molecular weight markers which were clearly detected in Red channel but did not yield strong signal in Green channel even with high very exposure.
