# Supplementary material for: Fortilin interacts with TGF-β1 and prevents TGF-β receptor activation
Source: Commun Biol. 2022 Feb 23;5:157. doi: 10.1038/s42003-022-03112-6 (PMC8866402; doi:10.1038/s42003-022-03112-6)
Supplement: Supplementary file 2 — Description of Additional Supplementary Files [file 42003_2022_3112_MOESM2_ESM.pdf]

Description of Additional Supplementary Files

File name: Supplementary Data 1

Description: All source data underlying the graphs and charts in the main figures have been uploaded as Supplementary Data in the Excel format.
