# Supplementary material for: Fortilin interacts with TGF-β1 and prevents TGF-β receptor activation
Source: Commun Biol. 2022 Feb 23;5:157. doi: 10.1038/s42003-022-03112-6 (PMC8866402; doi:10.1038/s42003-022-03112-6)
Supplement: Supplementary file 4 — Reporting Summary [file 42003_2022_3112_MOESM4_ESM.pdf]

Corresponding author(s): Ken Fujise
Last updated by author(s): 12/17/2021

Reporting Summary

Nature Portfolio wishes to improve the reproducibility of the work that we publish. This form provides structure for consistency and transparency in reporting. For further information on Nature Portfolio policies, see our Editorial Policies and the Editorial Policy Checklist.

Statistics

For all statistical analyses, confirm that the following items are present in the figure legend, table legend, main text, or Methods section.

n/a	Confirmed	
☐	☒	The exact sample size (*n*) for each experimental group/condition, given as a discrete number and unit of measurement
☐	☒	A statement on whether measurements were taken from distinct samples or whether the same sample was measured repeatedly
☐	☒	The statistical test(s) used AND whether they are one- or two-sided<br>*Only common tests should be described solely by name; describe more complex techniques in the Methods section.*
☒	☐	A description of all covariates tested
☒	☐	A description of any assumptions or corrections, such as tests of normality and adjustment for multiple comparisons
☐	☒	A full description of the statistical parameters including central tendency (e.g. means) or other basic estimates (e.g. regression coefficient) AND variation (e.g. standard deviation) or associated estimates of uncertainty (e.g. confidence intervals)
☐	☒	For null hypothesis testing, the test statistic (e.g. *F*, *t*, *r*) with confidence intervals, effect sizes, degrees of freedom and *P* value noted<br>*Give P values as exact values whenever suitable.*
☒	☐	For Bayesian analysis, information on the choice of priors and Markov chain Monte Carlo settings
☒	☐	For hierarchical and complex designs, identification of the appropriate level for tests and full reporting of outcomes
☒	☐	Estimates of effect sizes (e.g. Cohen's *d*, Pearson's *r*), indicating how they were calculated

Our web collection on statistics for biologists contains articles on many of the points above.

Software and code

Policy information about availability of computer code

Data collection	No custom algorithms or software was used. The following software was used for data collection:<br>(1) BLItz System (ForteBio) to obtain and analyze the biolayer interferometry data.<br>(2) BiaEvaluation Software (Biacore) to obtain and analyze the surface plasmon resonance data.
Data analysis	No custom algorithms or software was used. The following software was used for data collection:<br>(1) BLItz System (ForteBio) to obtain and analyze the biolayer interferometry data.<br>(2) BiaEvaluation Software (Biacore) to obtain and analyze the surface plasmon resonance data.<br>(3) ClusPro server was utilized to perform TGFbeta1-fortilin docking. The server has been extensively published including in Nature Protocol 2017;12 (2):255-278.

For manuscripts utilizing custom algorithms or software that are central to the research but not yet described in published literature, software must be made available to editors and reviewers. We strongly encourage code deposition in a community repository (e.g. GitHub). See the Nature Portfolio guidelines for submitting code & software for further information.

Data

Policy information about availability of data

All manuscripts must include a data availability statement. This statement should provide the following information, where applicable:

- Accession codes, unique identifiers, or web links for publicly available datasets
- A description of any restrictions on data availability
- For clinical datasets or third party data, please ensure that the statement adheres to our policy

All data are included in the paper and its extended data files.

Field-specific reporting

Please select the one below that is the best fit for your research. If you are not sure, read the appropriate sections before making your selection.

[x] Life sciences [ ] Behavioural & social sciences [ ] Ecological, evolutionary & environmental sciences

For a reference copy of the document with all sections, see nature.com/documents/nr-reporting-summary-flat.pdf

Life sciences study design

All studies must disclose on these points even when the disclosure is negative.

Sample size	Sample sizes were determined based on our prior experience or the reports using similar methods. No power analyses were used.
Data exclusions	No data were excluded unless outliers were identified and verified by the outlier test (Minitab, Version 20.1.3, State College, PA).
Replication	All cell-based experiments have been successfully repeated with comparable outcomes for at least twice.
Randomization	All experiments were biochemical or cell-based assays. No randomization was performed.
Blinding	The scientists were not blinded to allocation during experiments and readouts evaluation. All readouts from the experiments were predetermined, highly objective, and obtained according to the validated protocols.

Reporting for specific materials, systems and methods

We require information from authors about some types of materials, experimental systems and methods used in many studies. Here, indicate whether each material, system or method listed is relevant to your study. If you are not sure if a list item applies to your research, read the appropriate section before selecting a response.

Materials & experimental systems

n/a	Involved in the study	
[ ]	[x]	Antibodies
[ ]	[x]	Eukaryotic cell lines
[x]	[ ]	Palaeontology and archaeology
[x]	[ ]	Animals and other organisms
[x]	[ ]	Human research participants
[x]	[ ]	Clinical data
[x]	[ ]	Dual use research of concern

Methods

n/a	Involved in the study	
[x]	[ ]	ChIP-seq
[x]	[ ]	Flow cytometry
[x]	[ ]	MRI-based neuroimaging

Antibodies

Antibodies used

- Anti-fortilin (Abcam, Waltham, MA, USA; Clone EPR5540, ab133568; 1:2000 dilution) for Fig. S1B.
- Anti-fortilin (MRB International, Woburn, MA, USA; Catalog #: PM017; 1:1000 dilution) for Fig. S3.
- Anti-Gaussia luciferase (GLuc, NEB, Ipswich, MA, USA; Catalog #: E8023; 1:500 dilution)
- Anti-TGF-β1 (Abcam; Clone EPR18163, ab179695; 1:1000 dilution) for Western blot analysis of Fig. 1A and Fig. S1B.
- Anti-TGF-β1/2/3 (denoted anti-TGF-β)(R&D Systems, Minneapolis, MN, USA; Clone 1D11, MAB1835-100; 1:1000 dilution) for Western blot analysis of Fig. 1B.
- Anti-Flag (Sigma, St. Louis, MO, USA; Clone M2, F1804; 1:1000 dilution)
- Anti-His6 (Abcam; Clone HIS.H8, ab18184; 1:1000 dilution)
- Anti-GAPDH (Santa Cruz Biotechnology, Dallas, TX, USA; Clone 6C5, sc-32233; 1:10000 dilution)
- Anti-Strep tag (IBA Lifesciences, Göttingen, Germany; 2-1507-001; 1:1000 dilution)
- Anti-P-Smad3 (Rockland Immunochemicals, Limerick, PA, USA; Clone AF9F7, 600-401-919; 1:1000 dilution)

	• Anti-Smad3 (Abcam; Clone HIS.H8, ab75512; 1:1000 dilution)
Validation	All antibodies were purchased from the reputable vendors and used according to manufacturers' instructions.

Eukaryotic cell lines

Policy information about cell lines

Cell line source(s)	HEK-293 (ATCC CRL-1573) cells were obtained from American Type Culture Collection (ATCC, Manassas, VA, USA).<br>MFB-F11 cells were a kind gift from Dr. Tony Wyss-Coray (Stanford University, Stanford, CA, USA).
Authentication	Cell lines were obtained directly from A.T.C.C. or from the lab that originally generated the cell line and carefully maintained in the lab.
Mycoplasma contamination	All cells tested negative for mycoplasma.
Commonly misidentified lines<br>(See ICLAC register)	No commonly misidentified cell line was used.
